# Effects of human placenta cryopreservation on molecular characteristics of placental mesenchymal stromal cells

**DOI:** 10.3389/fbioe.2023.1140781

**Published:** 2023-04-13

**Authors:** Rūta Navakauskienė, Deimantė Žukauskaitė, Veronika Viktorija Borutinskaitė, Tetiana Bukreieva, Giedrė Skliutė, Elvina Valatkaitė, Aistė Zentelytė, Lina Piešinienė, Volodymyr Shablii

**Affiliations:** ^1^ Department of Molecular Cell Biology, Institute of Biochemistry, Life Sciences Center, Vilnius University, Vilnius, Lithuania; ^2^ Laboratory of Biosynthesis of Nucleic Acids, Department of Functional Genomics, Institute of Molecular Biology and Genetics, National Academy of Science, Kyiv, Ukraine; ^3^ Placenta Stem Cell Laboratory, Cryobank, Institute of Cell Therapy, Kyiv, Ukraine; ^4^ Nanodiagnostika, Ltd., Vilnius, Lithuania

**Keywords:** placenta, stromal cells, placenta-derivatives, tissue cryopreservation, animal study

## Abstract

Cryopreservation of placenta tissue for long-term storage provides the opportunity in the future to isolate mesenchymal stromal cells that could be used for cell therapy and regenerative medicine. Despite being widely used, the established cryopreservation protocols for freezing and thawing still raise concerns about their impact on molecular characteristics, such as epigenetic regulation. In our study, we compared the characteristics of human placental mesenchymal stromal cells (hPMSCs) isolated from fresh (native) and cryopreserved (cryo) placenta tissue. We assessed and compared the characteristics of native and cryo hPMSCs such as morphology, metabolic and differentiation potential, expression of cell surface markers, and transcriptome. No significant changes in immunophenotype and differentiation capacity between native and cryo cells were observed. Furthermore, we investigated the epigenetic changes and demonstrated that both native and cryo hPMSCs express only slight variations in the epigenetic profile, including miRNA levels, DNA methylation, and histone modifications. Nevertheless, transcriptome analysis defined the upregulation of early-senescence state-associated genes in hPMSCs after cryopreservation. We also evaluated the ability of hPMSCs to improve pregnancy outcomes in mouse models. Improved pregnancy outcomes in a mouse model confirmed that isolated placental cells both from native and cryo tissue have a positive effect on the restoration of the reproductive system. Still, the native hPMSCs possess better capacity (up to 66%) in comparison with cryo hPMSCs (up to 33%) to restore fertility in mice with premature ovarian failure. Our study demonstrates that placental tissue can be cryopreserved for long-term storage with the possibility to isolate mesenchymal stromal cells that retain characteristics suitable for therapeutic use.

## 1 Introduction

The discoidal placenta is a transient organ derived from maternal tissues (uterine decidua) and embryonic tissues (trophoblast–chorion). It is vital to effectively deliver nutrients and oxygen from the mother to the fetus (Sun et al., 2020). Even though most placentas are discarded after birth, they are routinely available, rich in the extracellular matrix, and a non-controversial source for the isolation of cells, being a valuable resource for regenerative medicine (Lobo et al., 2016). There are four distinct layers of the fetal placenta: chorionic mesenchymal, chorionic trophoblastic, amniotic epithelial, and amniotic mesenchymal (Parolini et al., 2007). The amniotic membrane is anti-inflammatory, anti-angiogenic, anti-fibrotic, and anti-microbial and reinforces epithelialization; thus, it can be used in regenerative medicine (Nejad et al., 2021); ophthalmology, especially for surgery and manufacturing of eye drops (Jirsova and Jones, 2017); periodontal surgery (Fénelon et al., 2018); sports medicine (Riboh et al., 2016), dermatology (Lo and Pope, 2009); treatment of osteoarthritis (Lopez-Martos et al., 2020) and so on. To avoid invasive procedures, mesenchymal stromal cells (MSCs) can be successfully retrieved from the amniotic mesenchymal placenta region (Aghayan et al., 2021). Using GMP practices and strict criteria for possible donors, placental tissue can be safely preserved and used on various occasions and for a wide range of procedures (Hahn et al., 2010).

Advanced techniques of cryopreservation, or storing samples at temperatures below freezing, have allowed the conservation of structurally sound living cells and biological tissues for later use (Pegg, 2007; Iussig et al., 2019). The most commonly used cryopreservation methods are slow-freezing and vitrification, but both have shortcomings (Crowley et al., 2021). While the procedure of cryopreservation is usually somewhat similar, there is a lack of standardization of methods of cryopreservation, which can distort the integrity and comparability of the results from various study groups (Dulugiac et al., 2015). Additionally, there is a risk of damaging cells with intracellular ice formation (Bahari et al., 2018), and viable cell yield after thawing is never 100%. The possibility of obtaining MSCs from cryopreserved placental tissue was demonstrated earlier (Shablii et al., 2012; Roselli et al., 2013; Nikulina et al., 2019). Nevertheless, there is no extensive analysis of proliferative activity, multipotency, transcriptome, and epigenetic alteration caused by cryopreservation of placental tissue. The influence of cryopreservation on umbilical cord tissue-derived MSCs has been investigated earlier (Roy et al., 2014; Dulugiac et al., 2015; Fong et al., 2016; Shivakumar et al., 2016). These studies have shown that MSCs isolated from cryopreserved placental or UC tissues maintain proliferative activity, stability of the karyotype, and differentiation potential into osteogenic, adipogenic, and chondrogenic lineages.

The establishment of public and private reserves of low-temperature placental tissue is a promising approach for long-term storage. For this, it is necessary to develop and standardize clinical-level protocols for producing MSC-based advanced therapy medical protocols and their quality control. It is currently created only for the widespread accumulation of umbilical cord tissue (Arutyunyan et al., 2018). It was shown that cryopreservation can lead to a decrease in the adhesion of MSCs to fibronectin, endothelial cells, engraftment potential in mouse lungs (Chinnadurai et al., 2014), and therapeutic potential in patients with acute graft versus host disease (Moll et al., 2014). Therefore, the effects of cryopreservation on hPMSCs, which would have therapeutic potential, need to be deeply evaluated.

In this research, we aimed to evaluate the effects of cryopreservation of human placenta tissue on placental mesenchymal stromal cells (hPMSCs). To do so, we compared the molecular parameters of hPMSCs isolated from fresh (native) and cryopreserved (cryo) placenta tissue of the same placenta samples. We analyzed the viability, proliferation, surface immunophenotype, differentiation potential, energy phenotype, and transcriptome of hPMSCs. Moreover, we looked at the genetic and epigenetic profile of hPMSCs, especially on DNA methylation and histone modification profile, miRs (miR-34a-3p, miR-29b-3p, and miR-145-5p) expression, and *in vivo* therapeutic potential for infertility treatment.

## 2 Materials and methods

### 2.1 Cultivation, viability, and proliferation assessment of placenta-derived stromal cells

This study and the consent procedure are approved by the Committee of Human Research of the Institute of Cell Therapy (#1-22) and by the Vilnius Regional Biomedical Research Ethics Committee (No. 158200-18/7-1049-550). Term placentas (*n* = 3; delivered after clinically normal pregnancies or Cesarean section) were collected from 23- to 36-year-old donors at 39–41 weeks of gestation in the Kyiv city maternity hospital #3. All donors provided written informed consent for sourcing and using their placentas for the approved study. The amnion was removed, and scissors were used to cut off an 8-g fragment of the chorionic plate and chorionic villus (3–7 mm thick). Half of the tissue was cryopreserved, and from another half of the fresh tissue, placenta-derived stromal cells were isolated, as described (Shablii et al. (2019).

For cryopreservation, Hank’s Balanced Salt solution (HBSS) (Sigma, Saint Louis, MO, United States) containing 10% DMSO (Sigma, Saint Louis, MO, United States) was added slowly for 15 min to small washed placental tissue fragments to obtain the final DMSO concentrations of 5%. The final volume of the sample prepared for cryopreservation contained placental tissue and cryopreservation medium in a ratio of 1:8. The samples were frozen in cryogenic vials following a unique program in a controlled-rate freezer (IceCube, Australia) as follows: from RT to −4°C with −1°C/min, held at −4°C to initiate ice formation, to −30°C with −1°C/min, kept at −30°C for 10 min, and then decreased to −140°C with 5°C/min. When the temperature reached −140°C, the cooling process (carried out in the freezer) was stopped, and the samples were transferred to liquid nitrogen for long-term storage (−196°C). Thawing was performed in a water bath at +38°C–40°C until the liquid phase (0°C) appeared, and then DMSO was gradually removed over 20 min by slowly adding HBSS to the tissue until a 5-fold dilution was reached. The thawed tissue was minced, and fragments were plated in culture flasks with complete growth media to obtain cell growth from explants.

The isolated cells were then transferred into T75 cm^2^ culture flasks and cultivated in DMEM/F12 growth medium (Gibco, Thermo Fisher Scientific, Waltham, MA, United States) supplemented with 15% FBS (Gibco, Thermo Fisher Scientific, Waltham, MA, United States), 100X MEM Non-Essential Amino Acid Solution and 1% penicillin (100 U/mL)–streptomycin (100 µg/mL) solution (Gibco, Thermo Fisher Scientific, Waltham, MA, United States). The growth conditions remained constant at 37°C, 5% CO_2_, with a humidified atmosphere throughout the cultivation period. Every 2–3 days, depending on the confluence (normally should be around 80%), cells were passaged, and their viability and proliferation were evaluated using the Trypan blue exclusion test. An equal volume of cells and 0.4% Trypan blue solution in 1X PBS was mixed, and using a hemocytometer with a Neubauer camera, the viability and total cell number were determined. By monitoring cell proliferation, the cumulative population doubling level (PDL) was calculated using the following formula:

$$PDL\left(X\right)=3.32\left(\log\left(TotalViableCellsatHarvest\right)/\left(TotalViableCellsatSeed\right)\right)+PDL\left(X-1\;Passage\right).$$

Moreover, the population doubling time of native and cryo cells was calculated according to the following formula and expressed as days:

$$Doubling\;Time=duration\times \log_{2}/\left(\log\left(FinalConcentration\right)-\log\left(InitialConcentration\right)\right).$$

### 2.2 CFU assay

A colony forming units (CFU) assay was carried out on passages 0, 2, and 6 for hPMSCs isolated from native and thawed tissue. At passage 0, 1 gram of fresh and thawed tissue (from three individual donors) was seeded into four T25 cm^2^ culture flasks, each in complete media, and cultured for 14 days. At passages 2 and 6, hPMSC suspension was diluted serially in alpha-MEM, and 1 mL of the last dilution corresponding to the concentration of 500 cells/mL was placed into a T25 cm^2^ tissue culture flask (Sarstedt, Germany) supplemented with 4 mL of complete medium to the final volume 5 mL and cultured for 14 days. Colonies were fixed with 4% PFA, stained with Romanowsky’s azure-eosin, and counted. Statistical analysis was performed using four independent values from each biological donor.

### 2.3 Assessment of cell energy phenotype

The metabolic potential and energy profile of placenta-derived native and cryo cells were determined using the Seahorse XFp Extracellular Flux Analyzer together with the Cell Energy Phenotype Test Kit (Agilent Technologies, Santa Clara, CA, United States) following the manufacturers’ guidelines. Briefly, this analysis simultaneously measures two energy-producing pathways—mitochondrial respiration and glycolysis—under baseline and induced stressed conditions, when the energy demand is increased. First, the oxygen consumption rate (OCR) and extracellular acidification rate (ECAR) were measured when the cells were at resting state. Then, to create a stressed phenotype, two stressor compounds, oligomycin and FCCP (carbonyl cyanide p-triflouromethoxyphenylhydrazone), which are the inhibitors of the electron transfer chain, were added to the media, and the OCR and ECAR were measured. After the test was completed, cells were collected and lysed with RIPA buffer (150 mM NaCl, 10 mM EDTA, pH 8.0, 10 mM Tris, pH 7.4, 0.1% SDS, 1% deoxycholate, 1% NP-40 in PBS, pH 7.6). Protein concentrations were evaluated using the Pierce Detergent Compatible Bradford Assay Kit (Thermo Fisher Scientific, Waltham, MA, United States) and an Infinite M200 Pro plate reader (Tecan, Switzerland). The OCR/ECAR ratio was calculated from normalized OCR and ECAR values, and metabolic potential was determined based on the percentage increase of stressed OCR over baseline OCR and stressed ECAR over baseline ECAR.

### 2.4 Flow cytometry analysis

Phenotypical characterization of native and cryo stromal cells by surface markers was performed by flow cytometry. For one assay, about 0.5× 10^5^ of cells were collected and centrifuged at 500 × *g* for 5 min. The growth medium was discarded, and the cells were washed twice using 1X PBS with 1% bovine serum albumin (BSA) and then resuspended in 50 µL PBS/1% BSA solution. Then, the appropriate amount of antibody against a specific surface marker was added, and the samples were incubated for 30 min in the dark at 4°C. After incubation, the cells were washed two times with PBS/1% BSA, then fixed with 2% paraformaldehyde (PFA) (Sigma-Aldrich, St. Louis, MO, United States), and finally analyzed with the Guava^®^easyCyte™ 8HT flow cytometer (Millipore, Burlington, MA, United States) using GuavaSoft™ 3.3 software. For this assay, the following antibodies were used: APC conjugated mouse anti-human antibodies: CD4, CD13, CD8, CD90, CD16, CD105, CD140b, SUSD2, CD117 (EXBIO, Vestec, Czech Republic), CD133, CD309, CD338, and SSEA4 (BioLegend, San Diego, CA, United States); FITC conjugated antibodies: CD9, CD73 (EXBIO, Vestec, Czech Republic), CD44 (Invitrogen, Thermo Fisher Scientific, Waltham, MA, United States), and CD45 (BD Pharmingen, San Jose, California, United States); Alexa Fluor^®^ 488 conjugated antibodies: CD14, CD31, CD146, HLA-ABC, and HLA-DR (BioLegend, San Diego, CA, United States); PE conjugated antibodies: CD3, CD15, CD19, CD144 (EXBIO, Vestec, Czech Republic), CD166, Notch1 (BioLegend, San Diego, CA, United States), and CD34 (Cell Signaling Technology, Danvers, MA, United States). The following isotype controls were chosen: APC conjugated: IgG1, IgG2a (EXBIO, Vestec, Czech Republic), IgG2b, and IgG1 (BioLegend, San Diego, CA, United States); FITC conjugated: IgG2a, IgG1 (EXBIO, Vestec, Czech Republic), IgG1, and IgG2a (BioLegend, San Diego, CA, United States); PE conjugated: IgM and IgG1 (EXBIO, Vestec, Czech Republic); PE conjugated: IgG1 (BioLegend, San Diego, CA, United States).

### 2.5 Differentiation assays

Placenta mesenchymal stromal cells were differentiated into adipogenic, osteogenic, and chondrogenic lineages. For adipogenic differentiation, cells were cultured to 80% confluence, and differentiation was induced using media consisting of DMEM (4.5 g/L glucose) supplemented with 10% FBS, 100 U/mL penicillin and 100 mg/mL streptomycin, 1 µM dexamethasone, 0.5 mM IBMX, and 60 µM indomethacin. For osteogenic differentiation, cells were cultured to 80% confluence as well, and differentiation was induced with DMEM (1 g/L glucose) media supplemented with 10% FBS, 100 U/mL penicillin and 100 mg/mL streptomycin, 0.1 µM dexamethasone, 10 mM β-glycerophosphate, and 50 µg/mL ascorbic acid. For chondrogenic differentiation, 1 × 10^5^ cells were seeded in a concentrated droplet of media (about 30 µL), and after a few hours, differentiation was induced using differentiation media consisting of DMEM (4.5 g/L glucose) supplemented with 100 U/mL penicillin and 100 mg/mL streptomycin, 0.1 µM dexamethasone, 50 µg/mL ascorbic acid, 1X insulin–transferrin–selenium (BioGems, Westlake Village, CA, United States), 10 ng/mL TGF-β3, 1 mM sodium pyruvate, and 0.35 mM proline. Cells were differentiated for 21 days with medium changes every 3–4 days. Adipogenic differentiation was confirmed by staining lipid droplets with Oil Red O solution (freshly diluted in distilled water at a ratio of 3:2), osteogenic differentiation was confirmed by staining calcium deposits with 2% Alizarin Red S solution (in deionized water), and chondrogenic differentiation was confirmed by staining glycosaminoglycans with 1% Alcian Blue (in 3% acetic acid). Differentiated cells were visualized using the EVOS XL Cell Imaging System (Thermo Fisher Scientific).

### 2.6 RNA isolation, RNA-seq, and RT-qPCR

Total RNA was extracted from hPMSCs using the Quick-DNA/RNA™ Miniprep Kit (Zymo Research, Irvine, CA, United States), and RNA purity and concentration were measured using the NanoDrop 2000 spectrophotometer (Thermo Fisher Scientific, United States). For RT-PCR, 500 ng of total RNA was converted to complementary DNA (cDNA) with the LunaScript^®^ RT SuperMix Kit (New England Biolabs, Ipswich, MA, United States) following the manufacturer’s recommendations. RT-qPCR was performed using Luna^®^ Universal qPCR Master Mix (New England Biolabs, Ipswich, MA, United States) and Rotor-Gene 6000 thermocycler with Rotor-Gene 6000 series software (Corbett Life Science, QIAGEN, Hilden, Germany). Gene expression was normalized to GAPDH and RPL13A (geometric mean). The list of primers and their sequences used in gene expression analysis is presented in Supplementary Table S1.

### 2.7 RNA-seq and bioinformatics analysis

The samples were prepared in biological triplicates. RNA was extracted using a NucleoSpin RNA isolation kit (Macherey–Nagel, 740955) according to the manufacturer’s protocol. RNA-seq libraries were prepared using the Agilent SureSelect Automated Strand-Specific RNA Library Prep, with polyA selection by Novogene Co., LTD (Beijing, China). Prepared libraries were sequenced on an Illumina HiSeq 2000 utilizing a paired-end 150-bp sequencing strategy (short-reads) and 20 M read pairs per sample. Raw data (raw reads) of fastq format were first processed through fastp software. In this step, clean data (clean reads) were obtained by removing reads containing the adapter, reads containing poly-N, and low-quality reads from raw data. At the same time, Q20, Q30, and GC content of the clean data was calculated. All the downstream analyses were based on clean data with high quality. Raw paired-end sequence reads were mapped to the human transcriptome (ensembl_homo_sapiens_grch38_p12_gca_000001405_27) using Hisat2 v2.0.5. Feature Counts v1.5.0-p3 was used to count the number of reads mapped to each gene. Then, Fragments Per Kilobase of transcript per Million of each gene was calculated based on the length of the gene and read count mapped to this gene. Differential expression analysis was performed using the DESeq2 R package (1.20.0). Genes with adjusted *p*-value < 0.05 and |log2(Fold Change)| > 0 were considered differentially expressed. The differentially expressed genes and selected categories of genes are listed in Supplementary Table S2. Clean data were deposited in the NCBI Sequence Read Archive (SRA). Enrichment analysis was performed using a graphical gene-set enrichment tool ShinyGO http://bioinformatics.sdstate.edu/go/ (Ge et al., 2019). Clean data were deposited in the NCBI Sequence Read Archive under BioProject accession number **PRJNA921741** (https://www.ncbi.nlm.nih.gov/bioproject/921741).

### 2.8 MicroRNA (miRNA) expression analysis

For miRNA expression analysis, RNA was reverse-transcribed using the TaqMan™ MicroRNA Reverse Transcription Kit and TaqMan™ MicroRNA Assay (Applied Bio-systems, Waltham, MA, United States). MicroRNA assays used for analysis were hsa-miR-34a-3p, hsa-miR-29b-3p, and hsa-miR-145-5p. miRNA expression levels were quantified using TaqMan™ MicroRNA Assay and TaqMan™ Universal PCR Master Mix II (Applied Biosystems, Waltham, MA, United States). The miRNA levels were normalized to RNU48. The relative expression of miRNA was calculated using the ΔΔ𝐶𝑡 method (compared to placenta cells isolated from fresh tissue).

### 2.9 Methylated DNA immunoprecipitation (MeDIP) and qPCR analysis

The Quick-DNA/RNA Miniprep Kit (Zymo Research, Irvine, CA, United States) was used for genomic DNA extraction from cell culture samples. For gDNA fragmentation, the Bioruptor^®^ Pico sonication device (Diagenode, Liege, Belgium) was used for seven cycles of 15 s on 90 s off to achieve DNA fragment size from 200 to 500 bp. Before immunoprecipitation, DNA samples were incubated at 95°C for 10 min to ensure the denaturation of the double-strand structure. A total of 320 ng DNA was mixed with 0.8 µL 1mg/1 mL anti-5-methylcytosine antibody (Eurogentec, Seraing, Belgium) and ZymoMag Protein A magnetic beads (Zymo Research, Irvine, CA, United States) and incubated at 37°C for 1 h on a rotator. Immunoprecipitated DNA was extracted magnetically using a magnetic tube rack. The qPCR was performed using Luna^®^ Universal qPCR Master Mix (New England BioLabs, Ipswich, MA, United States) with the Rotor-Gene 6000 system (Corbett Life Science, QIAGEN, Hilden, Germany). Sequences of gene promoter and exon-specific primers used for qPCR analysis are provided in Supplementary Table S3. The percentage of DNA input values for immunoprecipitated DNA samples was calculated according to the formulas:

$$∆Ct=\left(\left(Ct\left[InputDNA\right]–{\mathrm{Log}}_{2}10\right)-Ct\left[MeDIPDNA\right]\right).$$

$$\%\;ofInput=100\times 2^{∆Ct}.$$

### 2.10 Protein analysis using Western blot

Total protein extraction from native and cryo stromal cells was carried out according to the protocol described in Glemžaitė and Navakauskienė (2016). Briefly, cells from both groups were collected, washed twice with ice-cold PBS, and incubated with turbo nuclease (Jena Bioscience, Jena, Germany) on ice for 30 min. Then, the cells were suspended in SDS lysis buffer (125 mM Tris, pH 6.8, 4% SDS, 200 mM DTT, 20% glycerol, and traces of bromophenol blue), homogenized using a 26-G needle and incubated at 96°C for 5 min. After this, the samples were centrifuged at 20,000 × *g* for 15 min at 4°C, and the supernatant was collected and loaded into 7.5%–15% gradient polyacrylamide SDS/PAGE gel. After the proteins were fractioned, they were transferred onto PVDF membranes, and a Western blot was performed. The membrane was first incubated with the AdvanBlock-Chemi antibody antigen enhancing and blocking solution (Advansta, San Jose, CA, United States) and then with primary antibodies overnight at 4°C. To target specific proteins, the following anti-human antibodies were used: HDAC1 and HDAC2 (Santa Cruz Biotechnology, Dallas, TX, United States); H3K4me3, H4hyperAc, and H3K27me3 (Millipore, Burlington, MA, United States); H3K27Ac, H3K9Ac, and H3K9me3 (Cell Signaling Technology, Danvers, MA, United States). GAPDH, histone H4 (Abcam, Cambridge, United Kingdom), and histone H3 (Millipore, Burlington, MA, United States) were used as protein loading control for protein normalization. After incubation, the membrane was washed four times for 10 min with PBS-Tween 20 solution and incubated with secondary horseradish peroxidase-conjugated anti-mouse, anti-rabbit, and anti-goat antibodies for 1 h at RT. After washing, the chemiluminescent signal was detected using WesternBright ECL HRP substrate (Advansta, San Jose, CA, United States) and Chemi-Doc XRS + system with Image Lab Software (Bio-Rad Laboratories, Hercules, CA, United States). Subsequent calculations of the blot were performed using ImageJ software (NIH, United States).

### 2.11 Enzyme-linked immunosorbent assay—ELISA

ELISA was used to determine the secreted levels of IL-6, IL-8, and CCL-2. Secreted protein levels were measured using kits from R&D Systems, and all procedures were carried out according to the manufacturer’s instructions (R&D Systems, MN, United States). 96-well plates with samples were read with the spectrophotometer Infinite M200 Pro with i-control 1.5 software (Tecan). IL-6, IL-8, and CCL-2 values in media were normalized to cell number in culture at the time of collecting the media.

### 2.12 Animal study

The animal study protocol was approved by the ethics committee of the State Food and Veterinary Service of the Republic of Lithuania (protocol code No. G2-173 and date of approval 2019-05-20). This study was conducted in strict accordance with the recommendations in the “Guidelines for the Care and Use of Laboratory Animals of the National Institutes of Health.”

NOD.CB17-Prkdc ^scid^/Rj female mice (*n* = 50, 6 weeks of age; 18 g ± 0,5 g) were purchased from JANVIER LABS, France. All animals had free access to food (Scobis Uno, Mucedola) and water (sterile). Vaginal smears were used to monitor the estrus cycle. The experiment design of the animal study is summarized in Table 1. The animals were divided into two groups. One group was the control (untreated, *n* = 10): one part of the control mice was bred with male mice for 2–3 weeks (*n* = 5), and the pregnancy rate was observed. For the other part of the control mice, anti-Müllerian hormone (AMH) measurement was performed (*n* = 5). Another group (*n* = 40) was treated with an intraperitoneal injection of 30 mg/kg busulfan (Fresenius Kabi Deutschland GmbH, Germany) and 120 mg/kg cyclophosphamide (Baxter Oncology GmbH, Germany) to induce premature ovarian failure (POF), and they were observed for 1 week. Sterilized but without cell therapy mice (POF-group, *n* = 10) were chemotherapy controls (injected with saline only), of which one part of the treated mice were bred with male mice for 2–3 weeks (*n* = 5), and the pregnancy rate was observed. Another part was used for AMH measurement (*n* = 5). One week after the mice were injected with cyclophosphamide and busulfan, hPMSCs [native hPMSC (*n* = 15) or cryo hPMSCs (*n* = 15)] were transplanted into the ovary by intraovarian injection, as follows. Mice were anesthetized with inhalation of 1%–3% isoflurane (Isoflurin, Vetpharma, Spain), a small incision on the skin was made to access the ovary *via* the abdominal cavity, and the uterine horns were traced to identify the ovary. Then, approximately 20 µL of the cell suspension containing 0.5 × 10^6^ hPMSC (native or cryo) in PBS was injected into the left ovary (*n* = 30). One week after cell injection, animals were divided into two groups: one group [native hPMSCs (*n* = 12), and cryo hPMSCs (*n* = 12)] was bred with male mice for 2–3 weeks (the male to female ratio is 2:1), and the pregnancy rate was observed. The other group of mice [*n* = 6, native hPMSCs (*n* = 3), cryo hPMSCs (*n* = 3)] was euthanized, and blood was collected for AMH measurement by ELISA in triplicate according to a standard protocol and the manufacturers’ instructions. The pregnancy rate per group was calculated as the “number of pregnant mice/number of all mice in the group.”

**TABLE 1:** Experimental design of the animal study.

Group	Number of mice	Busulfan and cyclophosphamide treatment	Native hPMSC	Cryo hPMSC	Breeding	AMH level (without breeding)
Control (untreated)	10	No	No	No	Yes, *n* = 5	Yes, *n* = 5
Control POF	10	Yes, *n* = 10	No	No	Yes, *n* = 5	Yes, *n* = 5
POF + native hPMSC	15	Yes, *n* = 15	Yes, *n* = 15	No	Yes, *n* = 12	Yes, *n* = 3
POF + cryo hPMSC	15	Yes, *n* = 15	No	Yes, *n* = 15	Yes, *n* = 12	Yes, *n* = 3

### 2.13 Statistical analysis

Results are presented as mean ± standard deviation (S.D.) unless specified otherwise. The number of replicates for each experiment is provided in the Figure legends of reported results. Statistical analysis was calculated using GraphPad Prism 8.0.1 software (GraphPad Software, San Diego, CA, United States), and statistical significance for quantitative data was determined using the Mann–Whitney test and one-way ANOVA with Tukey’s *post-hoc* test. For qualitative data (pregnancy outcomes), the Chi-squared test was used. The significance level (α) was 0.05.

## 3 Results

### 3.1 Characterization of hPMSCs according to morphology, growth kinetics, and metabolism

**FIGURE 1:**
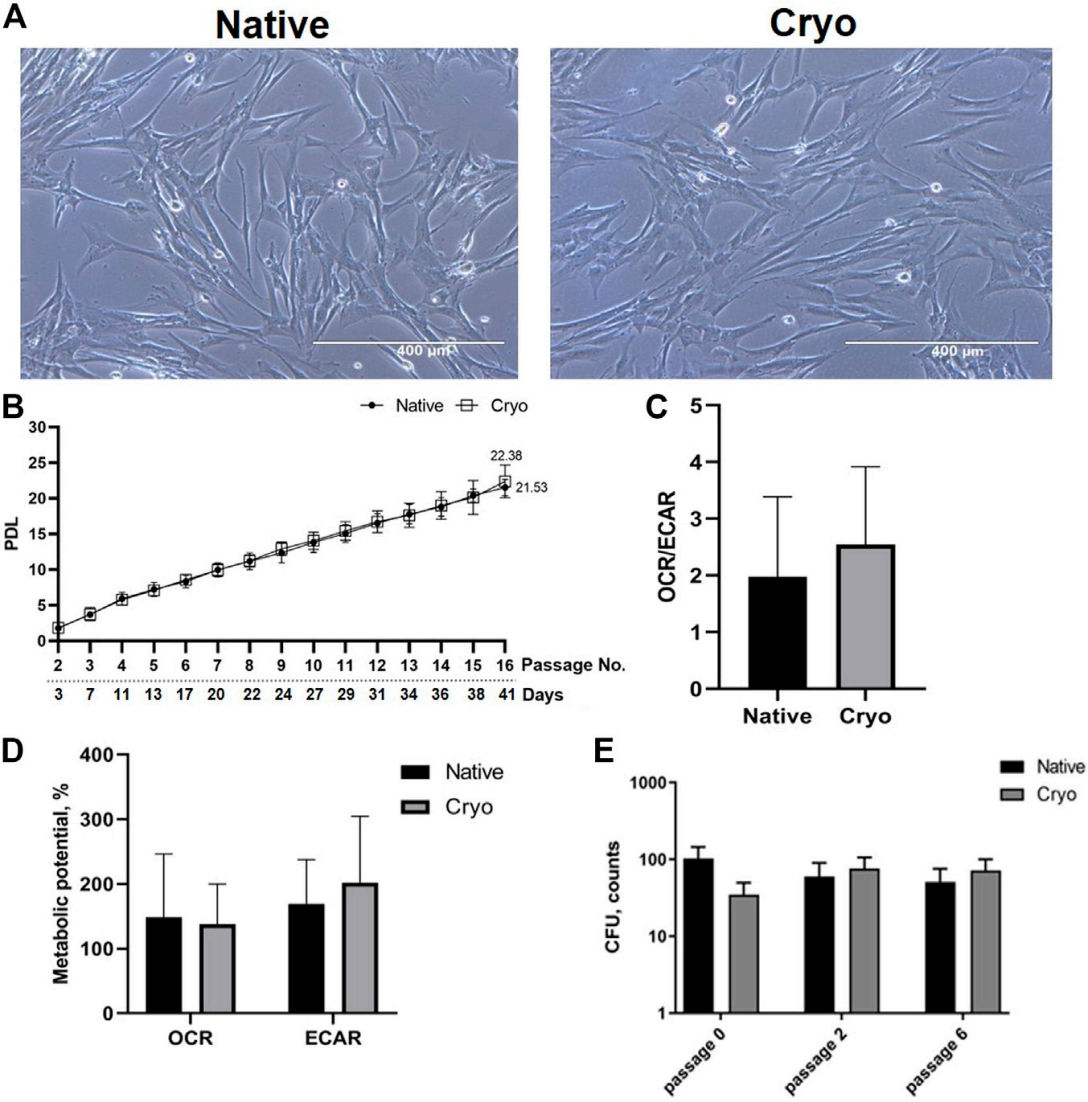
Characterization of hPMSCs by morphology, proliferation, and energetic phenotype. **(A)** Morphology of isolated and cultured cells derived from fresh placenta tissue (native) and from frozen tissue (cryo). Scale bar = 400 µm (10X objective). **(B)** Proliferation potential of native and cryo cells. Graph shows population doubling level (PDL), which is based on the initial and final concentration of cells, as well as passages and days in culture. Both cell populations were cultivated from the 2nd to 16th passage. Results are presented as mean ± S.D. (*n* = 6). **(C)** Analysis of the cellular energy consumption profile of native and cryo cells using Seahorse Extracellular Flux Analyzer. The ratio of oxygen consumption rate (OCR) and extracellular acidification rate (ECAR) was calculated under baseline conditions. Results are presented as mean ± S.D. (*n* = 6). **(D)** Assessment of metabolic potential changes in hPMSCs (native and cryo) under increased energy demand. OCR and ECAR parameters were measured separately under stressed conditions using Seahorse XFp Extracellular Flux Analyzer. All data were normalized to microgram of protein and presented as mean ± S.D. (*n* = 6). **(E)** Native and cryo cells did not differ in CFU activity, and data are presented as median and IQR (inter-quartile range), *n* = 4.

Isolated and cultured native and cryo stromal cells exhibited similar morphological properties (Figure 1A). The cells had elongated spindle-shaped morphology expressing a well-defined structure, with each cell harboring a single nucleus. No specific morphological differences were observed between native and cryo cells.

By further culturing stromal cells for a longer period of time, we determined the proliferation potential of the native and cryo cells (Figure 1B). As seen from the graph, the population doubling levels of the native and cryo stromal cells were almost exact. Native cells underwent a 21.53 population doubling and the cryo cells underwent a 22.38 population doubling. Cell doubling time for native cells was approximately 1.84 ± 0.49 days, while for cryo cells, it was around 1.91 ± 0.58 days, which means that both cell lines managed to maintain their population rates similarly during passaging.

Along with proliferation and cellular energy metabolism, more specifically, energy phenotype and metabolic potential of native and cryo stromal cells were determined (Figures 1C, D). We started our experiment by measuring the ratio of oxygen consumption rate (OCR) and extracellular acidification rate (ECAR) under baseline conditions. The decrease of oxygen concentration in the assay medium represents mitochondrial respiration of the cells, and the increase in proton concentration (or decrease in pH) in the assay medium describes glycolysis. As we see from the results, for energy production, placenta-derived native stromal cells tend to use mitochondrial respiration more than glycolysis (Figure 1C). The same tendency is seen with cryo stromal cells—under starting conditions, cryo cells mainly utilize mitochondrial respiration over glycolysis. There were no significant differences in energy consumption detected between native and cryo stromal cells.

Next, we determined the energy phenotype profile of placenta-derived native and cryo stromal cells under induced energy demand (Figure 1D). In the presence of stress-inducing compounds, native and cryo stromal cells displayed similar characteristics: they both tended to meet increased energy demand through glycolysis more than through oxidative phosphorylation. When comparing native and cryo cells with each other, it was detected that both cell groups utilize glycolysis at a similar level, showing that native and cryo cells act similarly when faced with extreme conditions. Also, we compared colony-forming units per 500 cells (CFU) in native and cryo cultures on passages 0, 2, and 6. No significant difference was revealed between native and cryo cells with each passage (Figure 1E).

### 3.2 Surface marker expression and differentiation potential of hPMSCs

**FIGURE 2:**
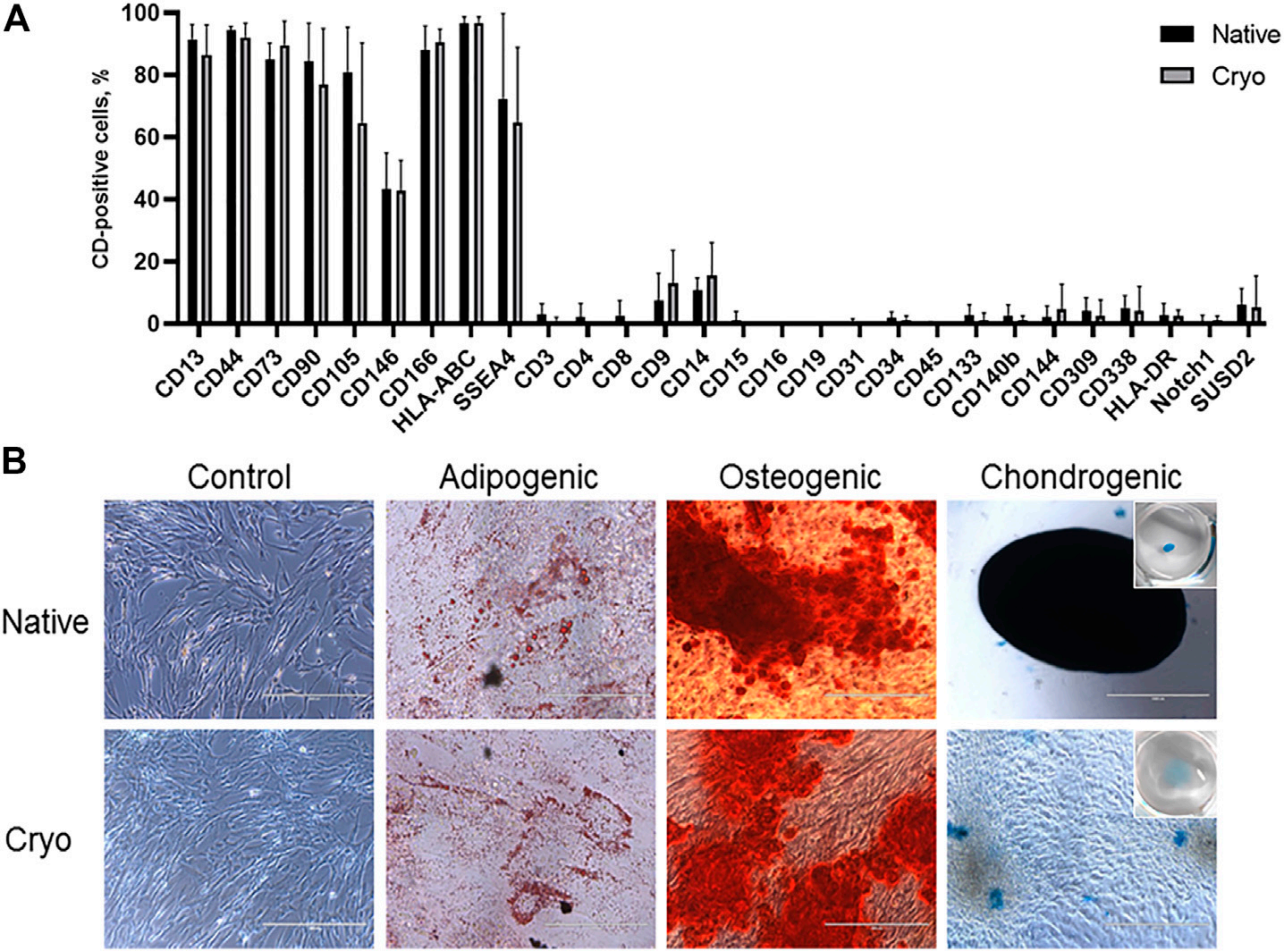
Evaluation of surface markers and differentiation potency of native and cryo hPMSCs. **(A)** Cell surface marker analysis of placenta-derived native and cryo stromal cells. Characterization of native and cryo cells was performed using CD13, CD44, CD73, CD90, CD105, CD146, CD166, HLA-ABC, SSEA4, CD3, CD4, CD8, CD9, CD14, CD15, CD16, CD19, CD31, CD34, CD45, CD133, CD140b, CD144, CD309, CD338, HLA-DR, Notch1, and SUSD2 markers. Analysis was performed using flow cytometry. Results are presented as mean ± S.D. (*n* = 6). **(B)** *In vitro* differentiation potential of native and cryo cells. Cells were differentiated into adipogenic (stained with Oil Red O), osteogenic (stained with Alizarin Red S), and chondrogenic (stained with Alcian Blue) lineages; representative images (scale bar = 400 μm, adipogenic differentiation = 100 μm, chondrogenic differentiation of native cells = 1,000 µm).

The fundamental part of cell characterization before any experimentation with new cell lines is surface marker analysis. Using flow cytometry, we tested native and cryo hPMSCs against 28 surface markers and determined their expression (Figure 2A). Surface markers associated with mesenchymal stem cells, such as CD13 (membrane alanyl aminopeptidase), CD44 (homing cell adhesion molecule), CD73 (5′-nucleotidase), CD146 (melanoma cell adhesion molecule), CD166 (activated leukocyte cell adhesion molecule), and HLA-ABC (major histocompatibility complex class I antigen), were highly expressed in both native and cryo cells at a comparable level. The expression of other mesenchymal markers CD90 (thymocyte differentiation antigen 1), CD105 (endoglin), and SSEA4 (stage-specific embryonic antigen-4) was differentially expressed in native and cryo stromal cells: in cryo cells, the expression was slightly decreased, although, it was not statistically significant. Both groups had low expression of endothelial and hematopoietic cell markers, including CD31 (platelet endothelial cell adhesion molecule), CD34 (hematopoietic progenitor cell antigen), CD117 (proto-oncogene c-kit), CD133 (prominin-1), and CD309 (fetal liver kinase 1). Surface markers for lymphoid cells, such as CD3, CD4, CD8, Notch1, and CD19, were identified as negative, expressing less than 5%. Similar tendencies can be seen in other immune cell markers, predominantly for myeloid cells: CD14, CD15, CD16, and HLA-DR.

Then, we evaluated the differentiation potential of native and cryo hPMSCs and determined that cells from both groups displayed differentiation potential toward three lineages of mesodermal fate, adipogenic, osteogenic, and chondrogenic, as determined by differentiation-specific staining with Oil Red O, Alizarin Red S, and Alcian Blue, respectively (Figure 2B). Both native and cryo cells showed similar differentiation potential toward adipogenic and osteogenic lineages. Only native cells could produce chondrogenic differentiation-specific three-dimensional cell spheroid with vibrant Alcian Blue staining. Meanwhile, cryo cells did not form a spheroid, and Alcian Blue staining was less intense (Figure 2B). Differentiated hPMSCs displayed significant upregulation of the chondrogenic differentiation-specific *COL2A1* (type II collagen, alpha 1 chain) gene in native and cryo cell groups, and significantly stronger upregulation was evident in cryo cells. During adipogenic differentiation, only native cells displayed significantly elevated expression of the PPARG (peroxisome proliferator-activated receptor gamma) gene, while osteogenic differentiation genes OPN (osteopontin) and ALP (alkaline phosphatase) showed a slight increase in both hPMSC groups, although this gene upregulation was not significant (Supplementary Figure S1).

### 3.3 Transcriptome analysis of native and cryo hPMSCs

**FIGURE 3:**
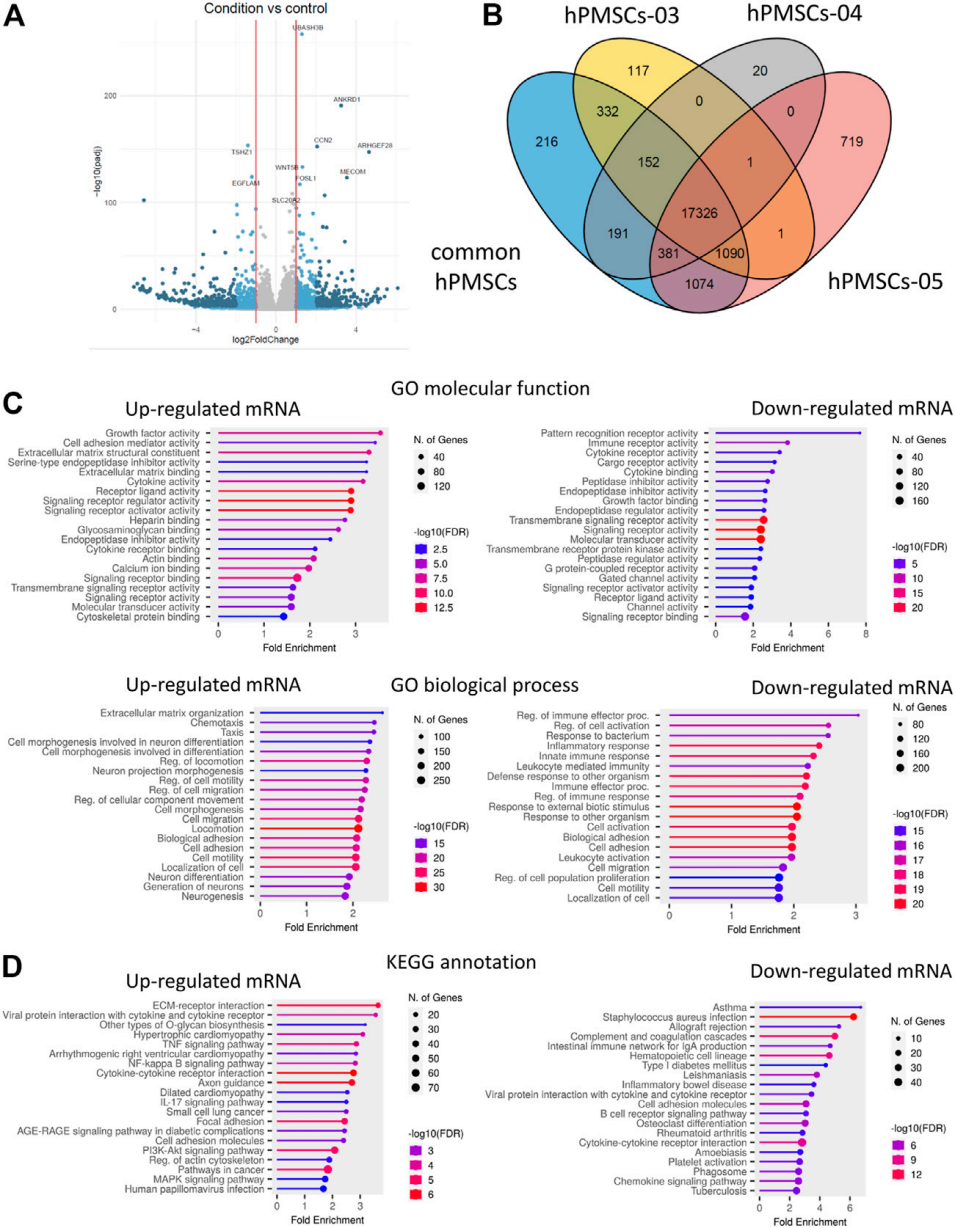
Transcriptome analysis of native and cryo hPMSCs. **(A)** Volcano plot for the genes differentially expressed between native and cryo hPMSCs. Darker colors correspond to lower p-adj values. **(B)** Venn diagram of independent comparison of numbers of differentially expressed genes; hPMSCs-03–05 represent three independent comparisons of native vs. cryo hPMSCs. **(C)** Gene Ontology overrepresentation analysis of the 3,514 genes falling into the cryopreservation-sensitive group. **(D)** KEGG annotation of differentially expressed genes in cryo hPMSCs. Experiments were performed in biological triplicates. The genes with p-adj < 0.05 were considered significant, log2Fold < −0.5—downregulated, log2Fold > 0.5—upregulated. FDR, false discovery rate.

To identify the genes with altered expression levels due to cryopreservation, we compared the transcriptome profiles of native and cryo hPMSCs. Results revealed that the 3,514 genes were differentially expressed between native and cryo hPMSCs (Figure 3A), 1,670 were upregulated, and 1,843 were downregulated in hPMSCs after cryopreservation. Heterogeneity between cell cultures is shown in the Venn diagram in Figure 3B. Gene ontology (GO) molecular function enrichment analysis of these upregulated genes revealed that growth factor activity, cell adhesion mediator activity, and extracellular matrix structural constituent are the most significantly overrepresented. GO biological process analysis showed that genes involved in extracellular matrix organization, chemotaxis, taxis, and cell morphogenesis were upregulated. Downregulated genes analyzed by GO molecular function showed that the most relevant categories were pattern recognition, immune, cytokine, and cargo receptor activities. GO biological process enrichment analysis of downregulated genes demonstrated that genes involved in the regulation of immune effector processing, regulation of cell activation, response to the bacterium, inflammatory response, and innate immune response were significantly underrepresented (Figure 3C). GO KEGG annotation revealed that inflammatory-associated genes involved in viral protein response, TNF, NF-kappa B, AGE-RAGE, cytokine–cytokine receptor, and IL-17 signaling pathways were upregulated in cryo hPMSCs compared to native ones. Furthermore, the ECM–receptor interaction was upregulated in KEGG annotation enrichment. Downregulated enriched categories analyzed by KEGG included genes associated with complement and coagulation cascades, *Staphylococcus* infection, leishmaniasis, and hematopoietic cell lineages. Interestingly, the highly differentially expressed genes enriched by the functional blocks related to antigen presentation, such as *HLA-F*, *HLA-DPB1*, *HLA-DOA*, *HLA-DPA1*, *HLA-DRB1*, and *HLA-DRA*, were strongly downregulated in cryo cells compared to native ones (Figure 3D).

**FIGURE 4:**
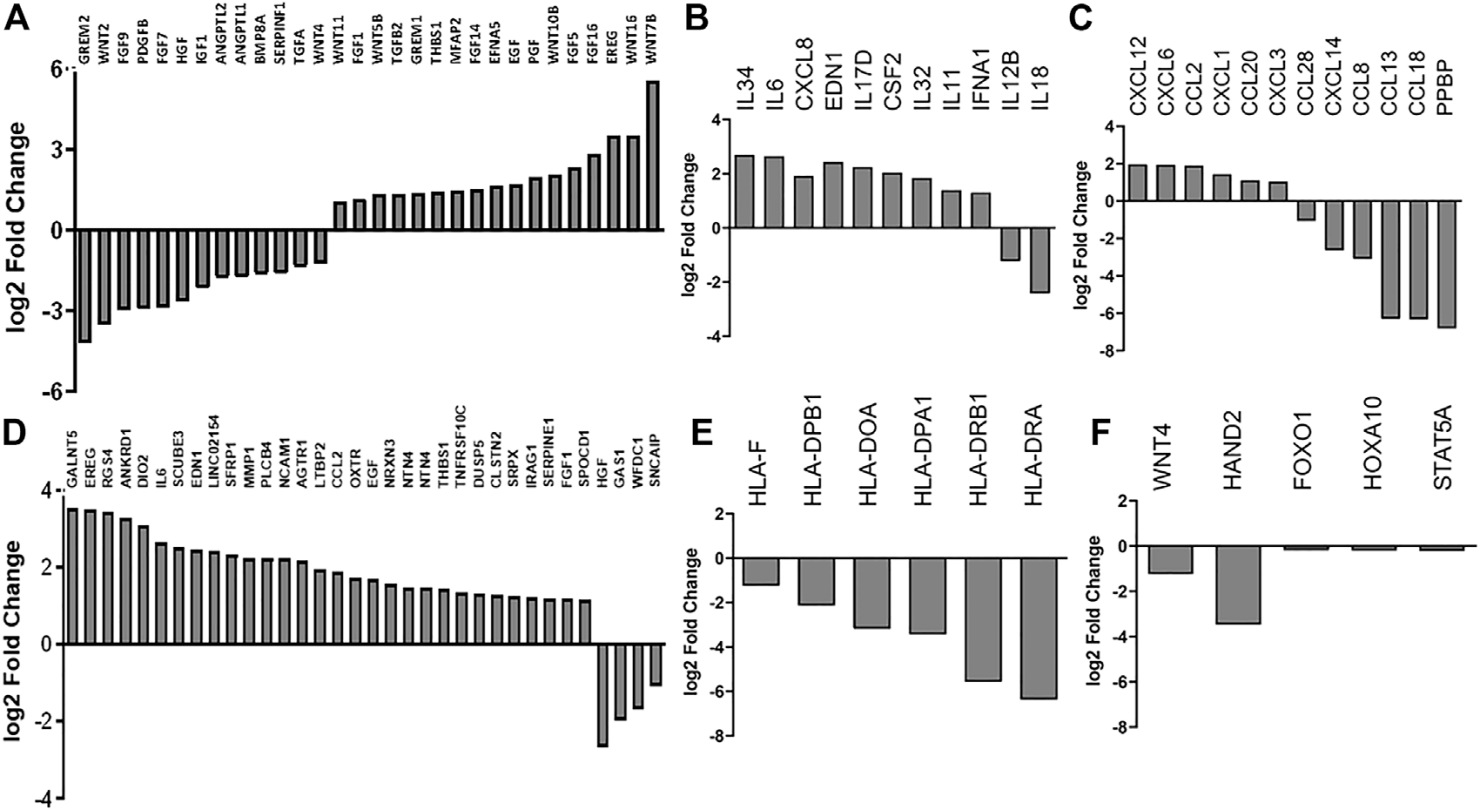
RNA-seq analysis of native and cryo stromal cells. The expression of highly differentially expressed genes enriched by the functional blocks related to **(A)** growth factors, **(B)** cytokines, **(C)** chemokines, **(D)** senescence-associated, **(E)** antigen-presenting, and **(F)** fertility-related genes. Data are represented as a fold change of the relative level of gene expression in cryo cells to native ones.

We were interested in showing the difference in cytokine and chemokine gene expression in native and cryo hPMSCs since the KEGG annotation showed the enrichment of cryo cells on inflammatory pathways, and the supposed main therapeutic effect of hPMSCs is due to their immunomodulatory properties (Figure 4). Among cytokines, cryo hPMSCs had a higher expression level of *IL34*, *IL6*, *CXCL8*, *EDN1*, *IL17D*, *CSF2*, *IL32*, *IL11*, and *IFNA1* and a lower expression level of *IL12B* and *IL18*. Chemokine genes demonstrated quite heterogeneous expression levels. Placenta-derived MSCs highly expressed chemokines such as *CXCL12*, *CXCL6*, *CCL2*, and *CXCL1* that were upregulated in cryo cells. The cryopreservation had a heterogeneous effect on the growth factor expression profile. Pro-inflammatory growth factors such as *THBS1*, *EFNA5*, *PGF*, *ANGPTL1* and *ANGPTL2*, and *EREG* were upregulated in cryo cells compared to native ones. At the same time, the anti-inflammatory factor *HGF* had a lower expression level in cryo cells, unlike *TGFB2* and *MFAP2*, which were upregulated.

We found an alteration in the expression of senescence-related genes in cryo hPMSCs in comparison with native hPMSCs. Thus, 31 upregulated hPMSC senescence-associated genes had increased levels of expression in cryo cells. The four highly downregulated differentially expressed hPMSC senescence-associated genes had lower expression levels in cryo compared to native cells. Therefore, the transcriptomic analysis revealed signs of senescence in cryo hPMSCs. Thus, the aforementioned data can suggest altered immunomodulating properties of cryo cells.

We also analyzed the secretion levels of IL-6, IL-8, and CCL-2 proteins in native and cryo placenta stromal cells using ELISA-based analysis. The results revealed that cells from both groups secreted IL-6, IL-8, and CCL-2 at comparable levels with no significant differences (Supplementary Figure S2), with IL-8 being the most abundant secreted protein of those tested.

### 3.4 Analysis of epigenetic regulation of placenta-derived stromal cells

**FIGURE 5:**
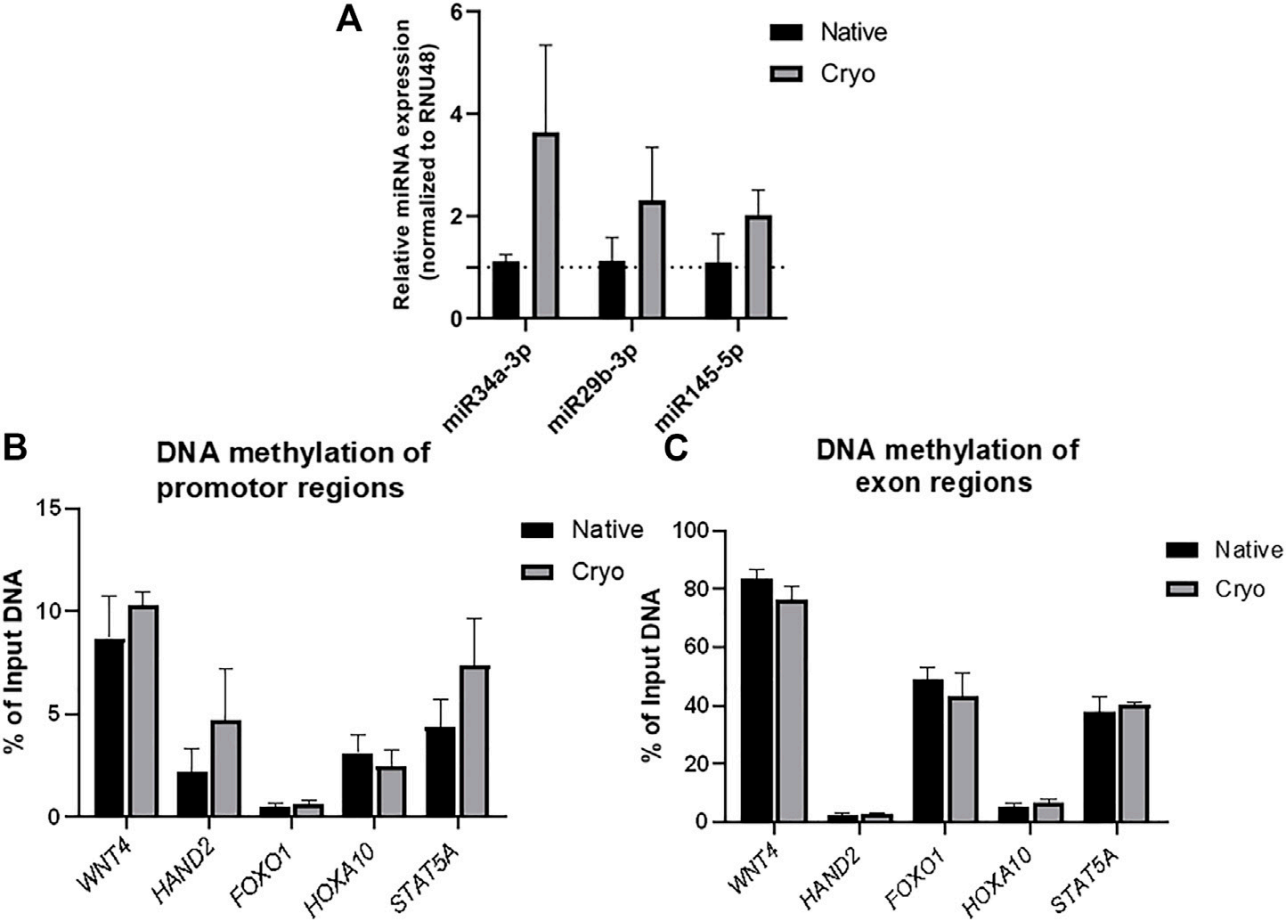
Epigenetic factor analysis of native and cryo hPMSCs. **(A)** miRNA expression evaluation of native and cryo hPMSCs by RT-qPCR. RNU48 was used as an internal control for miRNA level normalization. Results were calculated using the ΔΔCt method and represent changes from native cells. Results are shown as mean ± S.D. (*n* = 3). The levels of DNA methylation at specific gene (*WNT4*, *HAND2*, *FOXO1*, *HOXA10*, and *STAT5A*) promoters **(B)** and exon **(C)** regions were evaluated by performing methylated DNA immunoprecipitation (MeDIP) following qPCR analysis. IgG represents negative MeDIP test control. Results are shown as mean ± S.D. (𝑛 = 3) and represent the percentage of immunoprecipitated DNA amount to total DNA input.

miRNA analysis was performed to compare the expression levels of miR34a-3p, miR29b-3p, and miR145-5p between native and cryo hPMSCs. The analyzed miRNAs were selected as they contributed to reproductive biology regulation (Doridot et al., 2014; Kim et al., 2020; Wang and Li, 2020). miRNA analysis results revealed that levels of all three tested miRNAs, miR34a-3, miR29b-3p, and miR145-5p, were increased in placental cells isolated from cryopreserved placenta compared to cells derived from fresh tissue. However, changes were not significant (Figure 5A).

Furthermore, the levels of DNA methylation in *WNT4*, *HAND2*, *FOXO1*, *HOXA10*, and *STAT5A* promoters and exon regions were compared between native and cryo hPMSCs. No significant changes were observed in selected gene promoters (Figure 5B) or exon regions (Figure 5C) between tested groups. However, a tendency for a higher level of DNA methylation was detected in *HAND2* and *STAT5A* promotor sites of placental cells isolated from cryopreserved tissue.

### 3.5 Analysis of protein levels associated with epigenetic regulation

**FIGURE 6:**
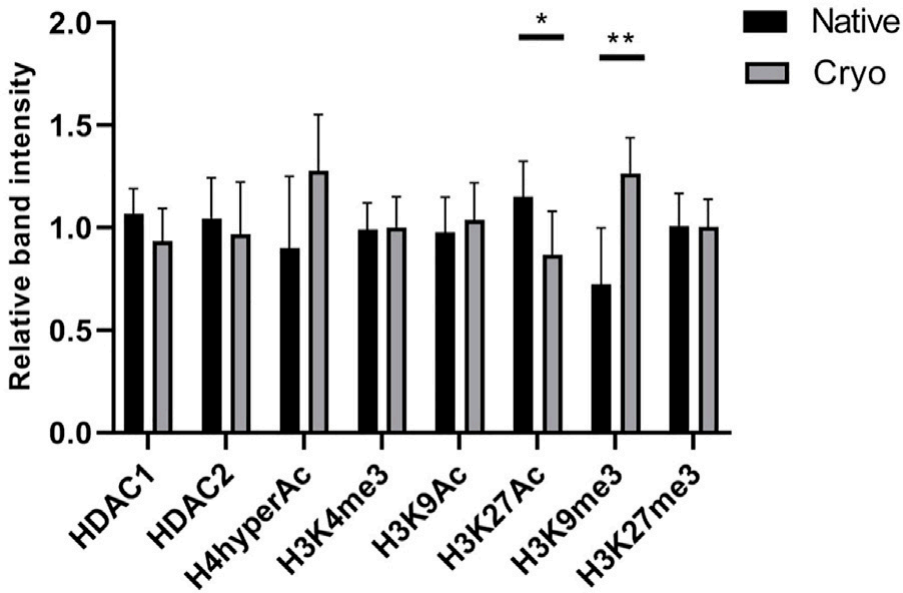
Assessment of protein levels in native and cryo stromal cells. Proteins associated with epigenetic regulation were evaluated using Western blot analysis, and relative band intensity was calculated with ImageJ software. The levels of HDAC1 and HDAC2 proteins were normalized to GAPDH and histone modifications according to H3 and H4 loading controls. Results are presented as mean ± S.D. (*n* = 6). Statistical analysis was calculated using the Mann–Whitney test, where *p ≤ 0.05; **p ≤ 0.01.

Proteins associated with epigenetic regulation were analyzed in native and cryo cells using an immunoassay (Western blot), and individual estimates of each protein band are presented in Supplementary Figure S3. The levels of histone deacetylase 1 and 2 (HDAC1 and HDAC2), which are involved in the modulation of chromatin architecture, were at a comparable level between native and cryo stromal cells (Figure 6(). Modified histones linked to transcriptionally active chromatin, including H4hyperAc, H3K4me3, and H3K9Ac, did not differ significantly. However, the level of histone modification in H3K27Ac was significantly higher in native cells than in cryo cells.

The level of another histone modification, H3K9me3, which is related to transcriptionally repressed chromatin, was determined to be lower in native cells than in cryo cells. This change was proven to be statistically significant. No major changes were observed in the levels of histone modification H3K27me3. Native and cryo stromal cells express this heterochromatin modification at a similar level.

### 3.6 Therapeutic effect of transplanted hPMSCs on fertility in a POF mouse model

To test the *in vivo* potential of hPMSCs of fresh (native) and cryopreserved placenta (cryo), we studied fertility reestablishment in the model mouse system. The estrus cycle stage was analyzed to confirm the POF induction, and 1 week after chemotherapy, the mice showed a disturbed estrous cycle, whereas normal mice maintained a normal 4- to 5-day estrous cycle (data not shown). Anti-Müllerian hormone (AMH), which is expressed by granulosa cells and its levels are significantly correlated with the reserve capacity of the ovary, was monitored in the blood of mice from all groups (control and treated). The highest level of AMH was observed in the control group, while in the POF group, the level of AMH was drastically reduced. After hPMSC administration, we observed increased AMH levels. However, it did not reach the untreated group level (Table 2). No drastic differences in AMH levels were observed between cryo and native groups.

**TABLE 2:** Fertility outcomes and AMH level in the POF mouse model.

Outcome	Control	POF model	Native hPMSC	Cryo hPMSC	*p*-value
Percentage of pregnancy	100%, *n* = 5	0%, *n* = 5	66%, *n* = 12	33%, *n* = 12	^a^ *p* = 0.1
Litter size (range min–max.)	6-12, *n* = 5	0, *n* = 5	5-8, *n* = 8	3-9, *n* = 4	^b^ *p* = 0.8
AMH level, pmol/L (range min–max.)	223-238, *n* = 5	17-25, *n* = 5	38.01–105.4, *n* = 3	49.42–97.26, *n* = 3	^c^ *p* = 0.03
					^d^ *p* = 0.06
					^e^ *p* = 0.7

^a^ Pregnancy outcome results of native and cryo cells compared using Chi-squared test.

^b^ Litter sizes compared between native and cryo groups analyzed using the Mann–Whitney test.

^c^ AMH levels between control and POF groups analyzed using the Mann–Whitney test.

^d^ AMH levels between POF- and hPMSC-treated groups (native or cryo) analyzed using the Mann–Whitney test.

^e^ AMH levels between native or cryo groups analyzed using the Mann–Whitney test.

**FIGURE 7:**
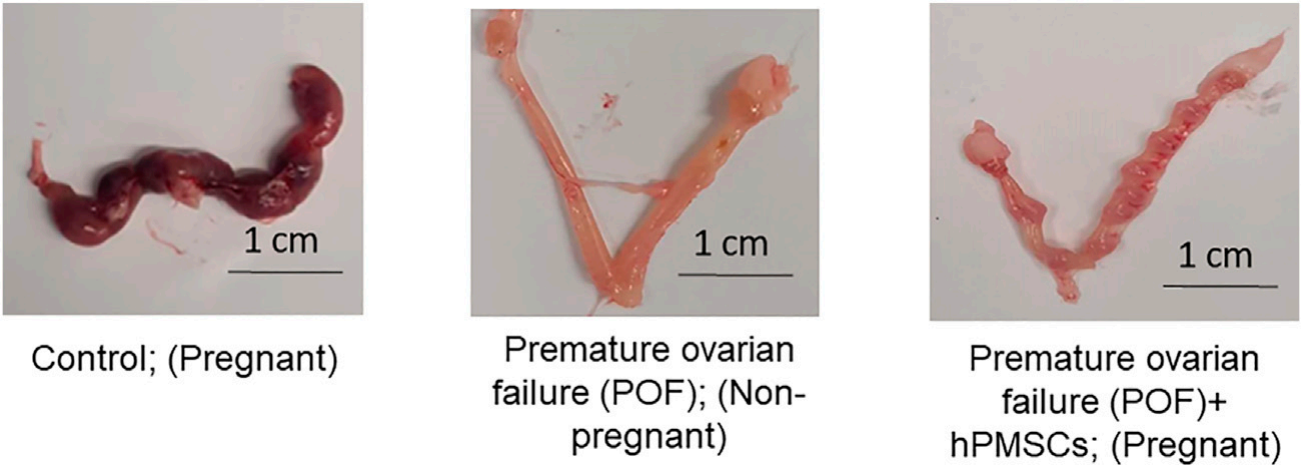
hPMSC effect on fertility in the POF mouse model. (Left image) Representative image of uterine horns of mice from the control group after breeding with male mice for 2 weeks. (Middle image) Representative image of uterine horns of mice from the POF group after breeding with male mice for 2 weeks. (Right image) Representative image of uterine horns of mice from the POF group after hPMSC intraovarian transplantation, after breeding with male mice for 2 weeks.

To analyze if the hPMSCs can have a positive (therapeutic) effect on fertility, we performed a breeding (fertility) test. Mice in three groups (control, POF, and POF after hPMSC treatment) were bred with male mice for 2 weeks, and the pregnancy rate (number of pregnant mice/all mice) in the groups was observed (Table 2; Supplementary Figure S4). The control mice (untreated) had the best pregnancy rate (100%), the highest AMH level (up to 238 pmol/L0), and the biggest litter size (mostly all mice had around 10 pups). The POF mice group had the lowest AMH level and a 0% pregnancy rate. The breeding test in the POF group was conducted for 4 months, and POF mice could not get pregnant during that period of time. In the fertility test after hPMSC treatment, our data revealed that both native and cryo hPMSCs have a positive effect on fertility in the POF mouse model (Table 2; Figure 7; Supplementary Figure S4). However, we observed differences in POF between native and cryo hPMSC treatment groups: the pregnancy rate in the native hPMSC group was 66% and that in cryo hPMSCs 33% (*p* = 0.1, according to Chi-square Test). Nevertheless, these data suggest that both native and cryo hPMSCs have a positive effect on fertility in the POF mice model, even at a different level.

## 4 Discussion

As cryopreservation became a widely used procedure for the long-term storage of cells and tissues for different applications, the safety and impact of cryopreservation on cellular properties and functions remain unclear (Chatterjee et al., 2016). Our study compared the molecular and therapeutic characteristics of human placental mesenchymal stromal cells (hPMSC) isolated from the freshly collected placenta (native) and cryopreserved tissue (cryo).

The cryopreservation approach is widely used for the long-term preservation of cells and tissues, especially in reproductive medicine. The biggest concern about cryopreservation is the cooling process itself, as it could cause intra- and inter-cellular ice crystal formation and damage cell integrity. To avoid it, slow freezing with cryoprotectants or vitrification is performed. Despite the established freezing protocols, the thawing process affects post-thaw cell survival and functionality properties (Chatterjee et al., 2017). However, the long-term effect of cells isolated from cryopreserved tissue was not yet properly investigated. On the other hand, Bahsoun et al. (2019) summarized the effect of cryopreservation on bone marrow mesenchymal stem cell viability, proliferation, morphology, and differentiation (Bahsoun et al., 2019). This systematic review shows that in most cases, the thawing of the cells did not negatively impact further cell proliferation, which is also observed in our study (Bahsoun et al., 2019). Furthermore, the morphology in most of the covered studies was not affected by cryopreservation, which corresponds with placental cryo cells compared to native cells observed in this study. It was shown that mesenchymal stromal cells isolated from cryopreserved human chorionic villi are genetically stable in long-term cultures up to passage 10 (Roselli et al., 2013). Although we demonstrated that viability, proliferation, differentiation, and energy phenotype did not differ between cells isolated from cryopreserved and fresh tissue, the alteration in the epigenetic profile and transcriptome was further investigated to recognize the cryopreservation impact on hPMSC properties.

We started our research by characterizing native and cryo hPMSCs on their surface marker expression using flow cytometry. We found that these cells expressed mesenchymal stem cell surface antigens (CD13, CD44, CD73, CD90, and CD105) at a high level and showed negative expression for endothelial and hematopoietic cell markers (CD14, CD31, CD34, CD45, CD117, and HLA-DR). Similar results were found by other authors, where a high expression of CD13, CD29, CD73, CD90, CD105, CD166, and HLA-ABC markers in placental mesenchymal stromal cell cultures was observed. The authors also confirmed hPMSCs to be non-hematopoietic and non-immunogenic since they were negative for CD14, CD31, CD34, CD45, and HLA-DR antigens (Tran et al., 2011; Vellasamy et al., 2012; Oliveira and Barreto-Filho, 2015). Similar to the results of other authors, the isolated hPMSCs displayed differentiation potential toward adipogenic, osteogenic, and chondrogenic lineage. Cells from fresh and cryopreserved placenta showed upregulation of differentiation-specific genes and displayed specific morphological changes attributed to each differentiation, like accumulation of lipid droplets for adipogenic, clustering of extracellular calcium deposits for osteogenic, and formation of cellular spheroids for chondrogenic differentiation (Tran et al., 2011; Choi et al., 2017). By assessing the growth kinetics of native and cryo stromal cells, we noticed that population doubling time was similar: for native cells, it was around 1.84 days (approx. 44 h), and for cryo cells, it was 1.91 days (46 h). According to Vellasamy et al. (2012), the doubling time of hPMSCs was 41 h on average, which also coincides with the study of Choi et al. (2017), where cells isolated from the whole placenta doubled their population in 41.57 ± 10.75 h (Vellasamy et al., 2012; Choi et al., 2017). These findings indicate that placenta-derived cells express typical mesenchymal stem cell surface markers, and the differentiation potential or proliferative activity was not affected by tissue cryopreservation.

The placenta is a highly complex organ connecting fetal and maternal systems. Many resources are required to sustain the needs of a developing placenta and to ensure that the necessary bioenergetic, signaling, proliferation, and differentiation functions are met. In this study, we measured the energy phenotype of native and cryo placenta-derived stromal cells using Seahorse Extracellular Flux Analyzer under baseline and stressed conditions. It is known that the protracted expansion of hPMSCs under this nutrient-rich environment induces a metabolic shift from glycolysis toward oxidative phosphorylation (Yuan et al., 2019; Yan et al., 2021). Nevertheless, we demonstrated that oxidative phosphorylation is the primary energy-producing pathway in hPMSCs, and no significant differences between native and cryo hPMSCs metabolic parameters were observed.

Epigenetic chromatin remodeling is essential for gene regulation in developing tissues such as the placenta. Histone modifications and DNA methylation are the primary targets when exploring epigenetic mechanisms (Apicella et al., 2019). MicroRNAs (miRNAs) are also considered epigenetic factors as they affect gene expression through post-transcriptional regulation, and investigating placental origin miRNAs revealed their importance in placental development and function (Mouillet et al., 2015). Recently, it was demonstrated that cryopreservation affects chromatin structure by damaging replication fork in replicating cells (Falk et al., 2018). Despite the previously described cryopreservation effect of cells, our study investigated how tissue cryopreservation impacts the epigenetics of hPMSCs. All tested miRNA (miR-34a-3p, miR-29b-3, and miR145-5p) levels showed an increasing tendency in cryopreserved tissue-derived hPMSCs compared to native hPMSCs. However, changes were not significant. Interestingly, the downregulation of miR-145-5p led to increased primordial follicle development and the number of primary and antral follicles (Kim et al., 2020). MiR-29b was upregulated in mural granulose cells (Andrei et al., 2019) and also reduced the expression of pro-proliferative genes and enhanced pro-autophagic gene expression while inhibiting granulosa cell apoptosis (Hilker, 2021). Downregulation of miR-34a resulted in the suppression of granulosa cell apoptosis in a porcine model and the activation of primordial follicle development in mice (Tu et al., 2014). The results of our study demonstrated that cryopreservation-associated stress could increase the levels of these miRNAs. However, the direct impact of miRNA changes should be further investigated as we did not observe a negative effect of cryopreservation on cell proliferation or other investigated properties.

Evaluation of DNA methylation of specific gene regions also did not reveal significant changes between native and cryo tissue isolated hPMSC groups. However, an increased tendency for DNA methylation in the HAND2 and STAT5A promotor sites in cryopreserved tissue-derived cells can be distinguished. Recently, cryopreservation was associated with an increase in ROS and oxidative stress, which could be linked with aberrant promoter hypermethylation and global hypomethylation (Reyes Palomares and Rodriguez-Wallberg, 2022). Furthermore, we demonstrated that in native and cryo stromal cells, histone modifications associated with transcriptionally active chromatin (H4hyperAc, H3K4me3, H3K9Ac, and H3K27Ac) were at a comparable level. Repressive markers, such as H3K27me3, did not differ between the groups, although the methylation of histone H3K9me3 decreased significantly in native cells. According to the observations of other authors, placental tissue generally has low DNA methylation levels (Lesch, 2021). Interestingly, this hypomethylation is especially distinguished in cytotrophoblasts, which remain hypomethylated throughout the whole period of gestation. It was revealed that this region remains transcriptionally repressed and has an H3K9me3 marker associated with it (Lesch, 2021). It should be noted that some modifications, such as DNA methylation and histone alterations, tend to be more sensitive to environmental shock factors (Chatterjee et al., 2017). According to a study by Yan et al. (2010), in which histone post-translational modifications in mouse oocytes were explored, histone H3K9 had increased methylation after these oocytes were vitrified (Yan et al., 2010). Although we did not detect significant changes in epigenetic regulation between native and cryo hPMSCs, the observed slight variations could suggest some freezing-caused epigenetic modifications in hPMSCs. However, it does not explain how these epigenetic changes could impact the molecular and therapeutic properties of cryo hPMSCs. Cryopreservation of placental tissue led to an alteration in the transcriptome of hPMSCs. Genes associated with various inflammatory signaling pathways (TNF, NF-kappa B, AGE-RAGE, cytokine–cytokine receptor, and IL-17 signaling pathways) had an increased expression level. Genes involved in extracellular matrix remodeling and cell differentiation, particularly in the neuronal direction, had a higher expression level in cryo hPMSCs. Extracellular matrix components and their receptors, as well as genes involved in extracellular matrix remodeling, had a higher expression level in senescent MSCs (Ghosh et al., 2020). In particular, it is known that during MSC aging, the expression of pro-inflammatory cytokines increases through the activation of the NF-kappa B signaling pathway (Chou et al., 2022; Lehmann et al., 2022). TNF alpha induces MSC senescence, and transplantation of senescent MSCs cannot successfully alleviate inflammatory diseases (Jung et al., 2022). Thus, it can be assumed that placental tissue cryopreservation could impact the therapeutic potential of hPMSCs. Also, cryopreservation of UC-MSCs could alter the expression of genes associated with differentiation, immunoregulation, regulation of cytokine production, cell migration, and others (Fu et al., 2020).

Chemotherapy with alkylating agents such as busulfan and cyclophosphamide can cause long-term damage effects, including rendering the recipient sterile (Park et al., 2013; Buigues et al., 2020). Busulfan, used for acute and chronic myeloid leukemia therapy, and cyclophosphamide, used for ovarian or breast cancer treatment, can directly or indirectly lead to apoptosis by inducing DNA damage, resulting in diminished ovarian reserves or primary ovarian insufficiency/failure (HalaMohamed et al., 2019; Kim and You, 2021). An increasing number of studies have revealed that stem/stromal cell transplantation is a very promising treatment for POF (Park et al., 2021). MSC transplantation can improve ovarian function in rats with chemotherapy-induced POF, at least partly through a paracrine mechanism (Ling et al., 2019; Malard et al., 2020). In this study, we explored the potential of hPMSCs of fresh and cryopreserved placenta to restore fertility in the POF mouse model. We have shown that both native and cryo hPMSCs can recover the fertility of chemotherapy-induced ovarian damage. However, higher pregnancy outcomes were achieved using native hPMSCs. We also demonstrated that hPMSC transplantation can increase the levels of AMH, an important fertility marker. Despite the calculated *p*-value (*p* = 0.06) slightly exceeding the significance level, our results demonstrate a clear tendency that native and cryo cells can improve the AMH level in the POF mouse model.

As our results suggest that native hPMSCs possess better therapeutic properties in the mouse model, we also showed the elevation in pro-inflammatory cytokine gene expression in hPMSCs isolated from cryopreserved placenta tissue. Furthermore, cryo cells had a higher expression level of chemokines CXCL12, CXCL6, CCL2, and CXCL1, which plays a key role in the chemotaxis of lymphocytes, monocytes, and neutrophils (Palomino and Marti, 2015). It should be known that the CD146^+^ MSC with lower levels of pro-inflammatory cytokines had stronger immunomodulatory properties and longer engraftment time after intravenous transplantation in mice with POF (Zhang et al., 2022). We observed the upregulation of several pro-inflammatory growth factors such as EFNA5 (Darling and Lamb, 2019), PGF (Li et al., 2017), and EREG (Borges et al., 2021) in cryo cells compared to native ones. On the other hand, anti-inflammatory growth factors THBS1, TGFB2 (Han et al., 2022), and MFAP2 (Gómez De Segura et al., 2021) had a higher level of expression in cryo hPMSCs. Previously, therapeutic effects of MSCs through THBS1-mediated induction of IL-10-producing B cells were shown on colitis (Liu et al., 2022). BDNF (Zhao et al., 2019; Kim and Hyun, 2022) plays a crucial role in supporting oocyte maturation. BMP4 initiates primordial follicle growth and prevents oocyte apoptosis in mice. Thus, we hypothesize that the downregulation of these growth factors in cryo cells compared to native cells could be the reason for the weaker impact on pregnancy rate in POF mice.

Furthermore, the comparative lower expression of angiogenesis-related genes ANGPTL1, ANGPTL2, PDGFB, and PDGFD in cryo cells could negatively impact the induction of blood vessel development and ovary restoration after POF. ANGPTL1 and ANGPTL2 were upregulated in the ovary after MSC injection (Santulli, 2014; Sasaki et al., 2015), whereas both genes had lower expression levels in cryo cells than in native cells in our study. PDGFB improved follicular survival as well as oocyte survival (Da Silva et al., 2018). PDGFB and PDGFD decreased in the follicular fluid of PCOS patients, while ovarian administration restored follicular development and angiogenesis in a rat model of polycystic ovary syndrome (Di Pietro et al., 2016). The lower relative expression levels of HGF in cryo cells compared to native ones could be the reason for the impaired effects on pregnancy rate since the hUC-MSC secretome promotes primordial follicle activation through HGF signaling (Mi et al., 2022). The deep RNA-seq analysis showed signs of senescence in cryo hPMSCs. Furthermore, the increased expression of pro-inflammatory secretory factors in cryo hPMSCs could indicate the early-senescence state, as was found previously in aged BM mesenchymal stromal cells (Medeiros Tavares Marques et al., 2017; Gnani et al., 2019). Thus, it seems likely that there is a connection between changes in the transcriptome, namely, in growth factor gene expression, upregulation of early-senescence state-associated genes, inflammatory-related genes, and downregulation of angiogenesis-related genes, with a decrease in the therapeutic potential of cryo hPMSCs in the POF of mice.

## 5 Conclusion

We demonstrated that both hPMSCs of fresh (native) and cryopreserved (cryo) placenta exhibit similar proliferation and energy profile, surface marker expression, and potential to multipotent differentiation with minor differences in miR expression and the alteration in expression of inflammatory-, angiogenesis-related, and early-senescence state-associated genes after cryopreservation. Injections of hPMSCs can restore fertility in a POF mouse model *via* the paracrine mechanism. In addition, native cells possess better capacity than cryo hPMSCs.

## Data Availability

The original contributions presented in the study are publicly available. These data can be found here: https://www.ncbi.nlm.nih.gov/bioproject/921741.

## References

[B1] Aghayan H. R. Payab M. Mohamadi-Jahani F. Aghayan S. S. Larijani B. Arjmand B. (2021). “GMP-compliant production of human placenta-derived mesenchymal stem cells,” in Stem cells and good manufacturing practices: Methods, protocols, and regulations. Editor Turksen K. (New York, NY: Springer US). 10.1007/7651_2020_282 32504292

[B2] Andrei D. Nagy R. A. Van Montfoort A. Tietge U. Terpstra M. Kok K. (2019). Differential miRNA expression profiles in cumulus and mural granulosa cells from human pre-ovulatory follicles. Microrna 8, 61–67. 10.2174/2211536607666180912152618 30207252 PMC6340152

[B3] Apicella C. Ruano C. S. M. Méhats C. Miralles F. Vaiman D. (2019). The role of epigenetics in placental development and the etiology of preeclampsia. Int. J. Mol. Sci. 20, 2837. 10.3390/ijms20112837 31212604 PMC6600551

[B4] Arutyunyan I. Fatkhudinov T. Sukhikh G. (2018). Umbilical cord tissue cryopreservation: A short review. Stem Cell Res. Ther. 9 (1), 236. 10.1186/s13287-018-0992-0 30219095 PMC6138889

[B5] Bahari L. Bein A. Yashunsky V. Braslavsky I. (2018). Directional freezing for the cryopreservation of adherent mammalian cells on a substrate. PLOS ONE 13, e0192265. 10.1371/journal.pone.0192265 29447224 PMC5813933

[B6] Bahsoun S. Coopman K. Akam E. C. (2019). The impact of cryopreservation on bone marrow-derived mesenchymal stem cells: A systematic review. J. Transl. Med. 17, 397. 10.1186/s12967-019-02136-7 31783866 PMC6883667

[B7] Borges J. P. Mekhail K. Fairn G. D. Antonescu C. N. Steinberg B. E. (2021). Modulation of pathological pain by epidermal growth factor receptor. Front. Pharmacol. 12, 642820. 10.3389/fphar.2021.642820 34054523 PMC8149758

[B8] Buigues A. Marchante M. Herraiz S. Pellicer A. (2020). Diminished ovarian reserve chemotherapy-induced mouse model: A tool for the preclinical assessment of new therapies for ovarian damage. Reprod. Sci. 27, 1609–1619. 10.1007/s43032-020-00191-w 32430713

[B9] Chatterjee A. Saha D. Glasmacher B. Hofmann N. (2016). Chilling without regrets. EMBO Rep. 17, 292–295. 10.15252/embr.201642069 26882559 PMC4772988

[B10] Chatterjee A. Saha D. Niemann H. Gryshkov O. Glasmacher B. Hofmann N. (2017). Effects of cryopreservation on the epigenetic profile of cells. Cryobiology 74, 1–7. 10.1016/j.cryobiol.2016.12.002 27940283

[B11] Chinnadurai R. Garcia M. A. Sakurai Y. Lam W. A. Kirk A. D. Galipeau J. (2014). Actin cytoskeletal disruption following cryopreservation alters the biodistribution of human mesenchymal stromal cells *in vivo* . Stem Cell Rep. 3 (1), 60–72. 10.1016/j.stemcr.2014.05.003 PMC4110775 25068122

[B12] Choi Y. S. Park Y. B. Ha C. W. Kim J. A. Heo J. C. Han W. J. (2017). Different characteristics of mesenchymal stem cells isolated from different layers of full term placenta. PLoS One 12, e0172642. 10.1371/journal.pone.0172642 28225815 PMC5321410

[B13] Chou L. Y. Ho C. T. Hung S. C. (2022). Paracrine senescence of mesenchymal stromal cells involves inflammatory cytokines and the NF-κB pathway. Cells 11, 3324. 10.3390/cells11203324 36291189 PMC9600401

[B14] Crowley C. A. Smith W. P. W. Seah K. T. M. Lim S.-K. Khan W. S. (2021). Cryopreservation of human adipose tissues and adipose-derived stem cells with DMSO and/or trehalose: A systematic review. Cells 10, 1837. 10.3390/cells10071837 34360005 PMC8307030

[B15] Da Silva R. F. Brito I. R. De Lima L. F. De Aguiar F. L. N. Rodrigues G. Q. Do Nascimento I. L. C. (2018). Platelet-derived growth factor-BB (PDGF-BB) improves follicular survival, oocyte and follicular diameters, in a dose-dependent manner, after the *in vitro* culture of goat preantral follicles enclosed in ovarian tissue fragments. Anim. Reprod. Ar. 14, 1095–1102. 10.21451/1984-3143-ar935

[B16] Darling T. K. Lamb T. J. (2019). Emerging roles for eph receptors and ephrin ligands in immunity. Front. Immunol. 10, 1473. 10.3389/fimmu.2019.01473 31333644 PMC6620610

[B17] Di Pietro M. Scotti L. Irusta G. Tesone M. Parborell F. Abramovich D. (2016). Local administration of platelet-derived growth factor B (PDGFB) improves follicular development and ovarian angiogenesis in a rat model of Polycystic Ovary Syndrome. Mol. Cell Endocrinol. 433, 47–55. 10.1016/j.mce.2016.05.022 27256152

[B18] Doridot L. Houry D. Gaillard H. Chelbi S. T. Barbaux S. Vaiman D. (2014). miR-34a expression, epigenetic regulation, and function in human placental diseases. Epigenetics 9 (1), 142–151. 10.4161/epi.26196 24081307 PMC3928177

[B19] Dulugiac M. Moldovan L. Zarnescu O. (2015). Comparative studies of mesenchymal stem cells derived from different cord tissue compartments - the influence of cryopreservation and growth media. Placenta 36, 1192–1203. 10.1016/j.placenta.2015.08.011 26343950

[B20] Falk M. Falková I. Kopečná O. Bačíková A. Pagáčová E. Šimek D. (2018). Chromatin architecture changes and DNA replication fork collapse are critical features in cryopreserved cells that are differentially controlled by cryoprotectants. Sci. Rep. 8, 14694. 10.1038/s41598-018-32939-5 30279538 PMC6168476

[B21] Fénelon M. Catros S. Fricain J. C. (2018). What is the benefit of using amniotic membrane in oral surgery? A comprehensive review of clinical studies. Clin. Oral Investig. 22, 1881–1891. 10.1007/s00784-018-2457-3 29682688

[B22] Fong C. Y. Subramanian A. Biswas A. Bongso A. (2016). Freezing of fresh wharton's jelly from human umbilical cords yields high post-thaw mesenchymal stem cell numbers for cell-based therapies. J. Cell. Biochem. 117 (4), 815–827. 10.1002/jcb.25375 26365815

[B23] Fu X. Xu B. Jiang J. Du X. Yu X. Yan Y. (2020). Effects of cryopreservation and long-term culture on biological characteristics and proteomic profiles of human umbilical cord-derived mesenchymal stem cells. Clin. Proteomics 17, 15. 10.1186/s12014-020-09279-6 32489333 PMC7247169

[B24] Ge S. X. Jung D. Yao R. (2019). ShinyGO: A graphical gene-set enrichment tool for animals and plants. Bioinformatics 36, 2628–2629. 10.1093/bioinformatics/btz931 PMC7178415 31882993

[B25] Ghosh D. Mejia Pena C. Quach N. Xuan B. Lee A. H. Dawson M. R. (2020). Senescent mesenchymal stem cells remodel extracellular matrix driving breast cancer cells to a more-invasive phenotype. J. Cell Sci. 133, jcs232470. 10.1242/jcs.232470 31932504 PMC6983709

[B26] Glemžaitė M. Navakauskienė R. (2016). Osteogenic differentiation of human amniotic fluid mesenchymal stem cells is determined by epigenetic changes. Stem Cells Int. 2016, 1. 10. 10.1155/2016/6465307 PMC5080506 27818691

[B27] Gnani D. Crippa S. Della Volpe L. Rossella V. Conti A. Lettera E. (2019). An early-senescence state in aged mesenchymal stromal cells contributes to hematopoietic stem and progenitor cell clonogenic impairment through the activation of a pro-inflammatory program. Aging Cell 18, e12933. 10.1111/acel.12933 30828977 PMC6516180

[B28] Gómez De Segura I. Ahechu P. Gómez-Ambrosi J. Rodríguez A. Ramírez B. Becerril S. (2021). Decreased levels of microfibril-associated glycoprotein (MAGP)-1 in patients with colon cancer and obesity are associated with changes in extracellular matrix remodelling. Int. J. Mol. Sci. 22, 8485. 10.3390/ijms22168485 34445187 PMC8395192

[B29] Hahn A. Thanos M. Reinhard T. Seitz B. Steuhl K. P. Meller D. (2010). Arbeitsrichtlin. *Der Ophthalmol.* 107, 1020–1031. 10.1007/s00347-010-2269-6 21088951

[B30] Hala H. Mohamed M. D. Badria F. Ahmed M. D. Fatma A. Abu Zahra M. D. (2019). The role of stem cells on the ovarian failure induced by busulfan in female albino rat. Med. J. Cairo Univ. 87, 1317–1330. 10.21608/mjcu.2019.53362

[B31] Han Y. Yang J. Fang J. Zhou Y. Candi E. Wang J. (2022). The secretion profile of mesenchymal stem cells and potential applications in treating human diseases. Signal Transduct. Target. Ther. 7, 92. 10.1038/s41392-022-00932-0 35314676 PMC8935608

[B32] Hilker R. (2021). The transcriptomic effects of MicroRNA-29b-3p in porcine granulosa cells. Guelph, Ontario: University of Guelph.

[B33] Iussig B. Maggiulli R. Fabozzi G. Bertelle S. Vaiarelli A. Cimadomo D. (2019). A brief history of oocyte cryopreservation: Arguments and facts. Acta Obstetricia Gynecol. Scand. 98, 550–558. 10.1111/aogs.13569 30739329

[B34] Jirsova K. Jones G. L. A. (2017). Amniotic membrane in ophthalmology: Properties, preparation, storage and indications for grafting—a review. Cell Tissue Bank. 18, 193–204. 10.1007/s10561-017-9618-5 28255771

[B35] Jung Y. H. Chae C. W. Chang H. S. Choi G. E. Lee H. J. Han H. J. (2022). Silencing SIRT5 induces the senescence of UCB-MSCs exposed to TNF-α by reduction of fatty acid β-oxidation and anti-oxidation. Free Radic. Biol. Med. 192, 1–12. 10.1016/j.freeradbiomed.2022.09.002 36096355

[B36] Kim J. You S. (2021). Extended adverse effects of cyclophosphamide on mouse ovarian function. BMC Pharmacol. Toxicol. 22, 3. 10.1186/s40360-020-00468-5 33413693 PMC7792169

[B37] Kim K.-H. Kim E.-Y. Kim G. J. Ko J.-J. Cha K. Y. Koong M. K. (2020). Human placenta-derived mesenchymal stem cells stimulate ovarian function via miR-145 and bone morphogenetic protein signaling in aged rats. Stem Cell Res. Ther. 11, 472. 10.1186/s13287-020-01988-x 33153492 PMC7643421

[B38] Kim M. Hyun S.-H. (2022). Neurotrophic factors in the porcine ovary: Their effects on follicular growth, oocyte maturation, and developmental competence. Front. Veterinary Sci. 9, 931402. 10.3389/fvets.2022.931402 PMC9399750 36032306

[B39] Lehmann J. Narcisi R. Franceschini N. Chatzivasileiou D. Boer C. G. Koevoet W. J. L. M. (2022). WNT/beta-catenin signalling interrupts a senescence-induction cascade in human mesenchymal stem cells that restricts their expansion. Cell. Mol. Life Sci. 79, 82. 10.1007/s00018-021-04035-x 35048158 PMC8770385

[B40] Lesch B. J. (2021). Epigenetic states in the human placenta: A singular epigenome for an exceptional tissue. Dev. Cell 56, 1211–1212. 10.1016/j.devcel.2021.04.011 33945779

[B41] Li X. Jin Q. Yao Q. Zhou Y. Zou Y. Li Z. (2017). Placental growth factor contributes to liver inflammation, angiogenesis, fibrosis in mice by promoting hepatic macrophage recruitment and activation. Front. Immunol. 8, 801. 10.3389/fimmu.2017.00801 28744285 PMC5504098

[B42] Ling L. Feng X. Wei T. Wang Y. Wang Y. Wang Z. (2019). Human amnion-derived mesenchymal stem cell (hAD-MSC) transplantation improves ovarian function in rats with premature ovarian insufficiency (POI) at least partly through a paracrine mechanism. Stem Cell Res. Ther. 10, 46. 10.1186/s13287-019-1136-x 30683144 PMC6347748

[B43] Liu J. Lai X. Bao Y. Xie W. Li Z. Chen J. (2022). Intraperitoneally delivered mesenchymal stem cells alleviate experimental colitis through THBS1-mediated induction of IL-10-competent regulatory B cells. Front. Immunol. 13, 853894. 10.3389/fimmu.2022.853894 35371051 PMC8971528

[B44] Lo V. Pope E. (2009). Amniotic membrane use in dermatology. Int. J. Dermatology 48, 935–940. 10.1111/j.1365-4632.2009.04173.x 19702975

[B45] Lobo S. E. Leonel L. C. P. C. Miranda C. M. F. C. Coelho T. M. Ferreira G. a. S. Mess A. (2016). The placenta as an organ and a source of stem cells and extracellular matrix: A review. Cells Tissues Organs 201, 239–252. 10.1159/000443636 27050810

[B46] Lopez-Martos R. Martin-Lozano G. Ocete-Perez R.-F. Gonzalez-Perez L.-M. Gutierrez-Perez J.-L. Infante-Cossio P. (2020). Application of human amniotic membrane in temporomandibular joint osteoarthritis. J. Craniofacial Surg. 31, e424–e426. 10.1097/scs.0000000000006424 32195845

[B47] Malard P. F. Peixer M. a. S. Grazia J. G. Brunel H. D. S. S. Feres L. F. Villarroel C. L. (2020). Intraovarian injection of mesenchymal stem cells improves oocyte yield and *in vitro* embryo production in a bovine model of fertility loss. Sci. Rep. 10, 8018. 10.1038/s41598-020-64810-x 32415089 PMC7229041

[B48] Medeiros Tavares Marques J. C. Cornélio D. A. Nogueira Silbiger V. Ducati Luchessi A. De Souza S. Batistuzzo De Medeiros S. R. (2017). Identification of new genes associated to senescent and tumorigenic phenotypes in mesenchymal stem cells. Sci. Rep. 7, 17837. 10.1038/s41598-017-16224-5 29259202 PMC5736717

[B49] Mi X. Jiao W. Yang Y. Qin Y. Chen Z.-J. Zhao S. (2022). HGF secreted by mesenchymal stromal cells promotes primordial follicle activation by increasing the activity of the PI3K-akt signaling pathway. Stem Cell Rev. Rep. 18, 1834–1850. 10.1007/s12015-022-10335-x 35089464 PMC9209380

[B50] Moll G. Alm J. J. Davies L. C. von Bahr L. Heldring N. Stenbeck-Funke L. (2014). Do cryopreserved mesenchymal stromal cells display impaired immunomodulatory and therapeutic properties? Stem cells Dayt. Ohio) 32 (9), 2430–2442. 10.1002/stem.1729 PMC4381870 24805247

[B51] Mouillet J. F. Ouyang Y. Coyne C. B. Sadovsky Y. (2015). MicroRNAs in placental health and disease. Am. J. Obstet. Gynecol. 213, S163–S172. 10.1016/j.ajog.2015.05.057 26428496 PMC4592520

[B52] Nejad A. R. Hamidieh A. A. Amirkhani M. A. Sisakht M. M. (2021). Update review on five top clinical applications of human amniotic membrane in regenerative medicine. Placenta 103, 104–119. 10.1016/j.placenta.2020.10.026 33120046

[B53] Nikulina V. Kuchma M. Bukrejeva T. Zahanich I. Kyryk V. Lobintseva G. (2019). Cryopreservation of placenta tissue allows isolating viable mesenchymal and hematopoietic stem cells. Cytotherapy 21, S78–S79. 10.1016/j.jcyt.2019.03.485

[B54] Oliveira M. S. Barreto-Filho J. B. (2015). Placental-derived stem cells: Culture, differentiation and challenges. World J. Stem Cells 7, 769–775. 10.4252/wjsc.v7.i4.769 26029347 PMC4444616

[B55] Palomino D. C. Marti L. C. (2015). Chemokines and immunity. Einstein (Sao Paulo) 13, 469–473. 10.1590/s1679-45082015rb3438 26466066 PMC4943798

[B56] Park H. S. Chugh R. M. Elsharoud A. Ulin M. Esfandyari S. Aboalsoud A. (2021). Safety of intraovarian injection of human mesenchymal stem cells in a premature ovarian insufficiency mouse model. Cell Transpl. 30 (963689720988502), 096368972098850. 10.1177/0963689720988502 PMC7894598 33593078

[B57] Park M.-R. Choi Y.-J. Kwon D.-N. Park C. Bui H.-T. Gurunathan S. (2013). Intraovarian transplantation of primordial follicles fails to rescue chemotherapy injured ovaries. Sci. Rep. 3, 1384. 10.1038/srep01384 23463338 PMC3589785

[B58] Parolini O. Alviano F. Bagnara G. P. Bilic G. Bühring H.-J. Evangelista M. (2007). Concise review: Isolation and characterization of cells from human term placenta: Outcome of the first international workshop on placenta derived stem cells. Stem Cells 26, 300–311. 10.1634/stemcells.2007-0594 17975221

[B59] Pegg D. E. (2007). “Principles of cryopreservation,” in Cryopreservation and freeze-drying protocols. Editors Day J. G. Stacey G. N. (Totowa, NJ: Humana Press).

[B60] Reyes Palomares A. Rodriguez-Wallberg K. A. (2022). Update on the epigenomic implication of embryo cryopreservation methods applied in assisted reproductive technologies with potential long-term health effects. Front. Cell Dev. Biol. 10, 881550. 10.3389/fcell.2022.881550 35573677 PMC9096028

[B61] Riboh J. C. Saltzman B. M. Yanke A. B. Cole B. J. (2016). Human amniotic membrane–derived products in sports medicine:basic science, early results, and potential clinical applications. Am. J. Sports Med. 44, 2425–2434. 10.1177/0363546515612750 26585668

[B62] Roselli E. A. Lazzati S. Iseppon F. Manganini M. Marcato L. Gariboldi M. B. (2013). Fetal mesenchymal stromal cells from cryopreserved human chorionic villi: Cytogenetic and molecular analysis of genome stability in long-term cultures. Cytotherapy 15, 1340–1351. 10.1016/j.jcyt.2013.06.019 24094486

[B63] Roy S. Arora S. Kumari P. Ta M. (2014). A simple and serum-free protocol for cryopreservation of human umbilical cord as source of Wharton's jelly mesenchymal stem cells. Cryobiology 68 (3), 467–472. 10.1016/j.cryobiol.2014.03.010 24704519

[B64] Santulli G. (2014). Angiopoietin-like proteins: A comprehensive look. Front. Endocrinol. (Lausanne) 5, 4. 10.3389/fendo.2014.00004 24478758 PMC3899539

[B65] Sasaki Y. Ohta M. Desai D. Figueiredo J.-L. Whelan M. C. Sugano T. (2015). Angiopoietin like protein 2 (ANGPTL2) promotes adipose tissue macrophage and T lymphocyte accumulation and leads to insulin resistance. PLOS ONE 10, e0131176. 10.1371/journal.pone.0131176 26132105 PMC4489192

[B66] Shablii V. Kuchma M. Kyryk V. Onishchenko G. Tsupykov O. Klymenko P. (2012). Mesenchymal stromal cells from native and cryopreserved human placenta: Phenotype, multipotency and *in vivo* migration potential. Problems Cryobiol. Cryomedicine 22 (2), 157–160.

[B67] Shablii V. Kuchma M. Svitina H. Skrypkina I. Areshkov P. Kyryk V. (2019). High proliferative placenta-derived multipotent cells express cytokeratin 7 at low level. BioMed Res. Int. 2019, 1. 13. 10.1155/2019/2098749 PMC6662495 31392209

[B68] Shivakumar S. B. Bharti D. Subbarao R. B. Jang S. J. Park J. S. Ullah I. (2016). DMSO- and serum-free cryopreservation of wharton's jelly tissue isolated from human umbilical cord. J. Cell. Biochem. 117 (10), 2397–2412. 10.1002/jcb.25563 27038129 PMC5094545

[B69] Sun C. Groom K. M. Oyston C. Chamley L. W. Clark A. R. James J. L. (2020). The placenta in fetal growth restriction: What is going wrong? Placenta 96, 10–18. 10.1016/j.placenta.2020.05.003 32421528

[B70] Tran T. C. Kimura K. Nagano M. Yamashita T. Ohneda K. Sugimori H. (2011). Identification of human placenta-derived mesenchymal stem cells involved in re-endothelialization. J. Cell Physiol. 226, 224–235. 10.1002/jcp.22329 20658518

[B71] Tu F. Pan Z. X. Yao Y. Liu H. L. Liu S. R. Xie Z. (2014). miR-34a targets the inhibin beta B gene, promoting granulosa cell apoptosis in the porcine ovary. Genet. Mol. Res. 13, 2504–2512. 10.4238/2014.January.14.6 24446339

[B72] Vellasamy S. Sandrasaigaran P. Vidyadaran S. George E. Ramasamy R. (2012). Isolation and characterisation of mesenchymal stem cells derived from human placenta tissue. World J. Stem Cells 4, 53–61. 10.4252/wjsc.v4.i6.53 22993662 PMC3443712

[B73] Wang L. Li Y. (2020). MiR-29b-3p affects growth and biological functions of human extravillous trophoblast cells by regulating bradykinin B2 receptor. Archives Med. Sci. AMS 18 (2), 499–522. 10.5114/aoms.2019.91512 PMC8924841 35316906

[B74] Yan L. Y. Yan J. Qiao J. Zhao P. L. Liu P. (2010). Effects of oocyte vitrification on histone modifications. Reprod. Fertil. Dev. 22, 920–925. 10.1071/rd09312 20591326

[B75] Yan W. Diao S. Fan Z. (2021). The role and mechanism of mitochondrial functions and energy metabolism in the function regulation of the mesenchymal stem cells. Stem Cell Res. Ther. 12, 140. 10.1186/s13287-021-02194-z 33597020 PMC7890860

[B76] Yuan X. Logan T. M. Ma T. (2019). Metabolism in human mesenchymal stromal cells: A missing link between hMSC biomanufacturing and therapy? Front. Immunol. 10, 977. 10.3389/fimmu.2019.00977 31139179 PMC6518338

[B77] Zhang L. Sun Y. Zhang X.-X. Liu Y.-B. Sun H.-Y. Wu C.-T. (2022). Comparison of CD146 +/− mesenchymal stem cells in improving premature ovarian failure. Stem Cell Res. Ther. 13, 267. 10.1186/s13287-022-02916-x 35729643 PMC9209844

[B78] Zhao X. Du F. Liu X. Ruan Q. Wu Z. Lei C. (2019). Brain-derived neurotrophic factor (BDNF) is expressed in buffalo (Bubalus bubalis) ovarian follicles and promotes oocyte maturation and early embryonic development. Theriogenology 130, 79–88. 10.1016/j.theriogenology.2019.02.020 30877846

