# Supplementary material for: Effects of human placenta cryopreservation on molecular characteristics of placental mesenchymal stromal cells
Source: Front Bioeng Biotechnol. 2023 Apr 13;11:1140781. doi: 10.3389/fbioe.2023.1140781 (PMC10133466; doi:10.3389/fbioe.2023.1140781)
Supplement: Supplementary file 2 [file Table1.DOCX]

Supplementary Material

Effects of Human Placenta Cryopreservation on Molecular Characteristics of Placenta Mesenchymal Stromal Cells

Rūta Navakauskienė^1^*, Deimantė Žukauskaitė^1^, Veronika Viktorija Borutinskaitė^1^, Tetiana Bukreieva^2,3^, Giedrė Skliutė^1,4^, Elvina Valatkaitė^1^, Aistė Zentelytė^1^, Lina Piešinienė^4^, Volodymyr Shablii^2,3^*

*** Correspondence:** Rūta Navakauskienė: [ruta.navakauskiene@bchi.vu.lt](mailto:ruta.navakauskiene@bchi.vu.lt); Volodymyr Shablii: [shablii@stemcellclinic.com.ua](mailto:shablii@stemcellclinic.com.ua)

**Supplementary Figure 1.** Gene expression analysis of differentiated Native (*n*=3) and Cryo (*n*=3) hPMSCs towards adipogenic (*PPARG*), osteogenic (*OPN, ALP*), and chondrogenic (*COL2A1*) lineages. RT-qPCR data is represented as relative fold change over undifferentiated control, normalized for the housekeeping genes *GAPDH* and *RPL13A*; values are indicated as mean ± SD. Statistical analysis was calculated using one-way ANOVA with Tukey’s *post hoc* test, where ** *p* ≤ 0.01, *****p* ≤ 0.0001.

**Supplementary Figure 2.** Secretion levels of IL-6, IL-8, and CCL2 proteins in Native (*n*=2) and Cryo (*n*=2) hPMSCs. Secretion of proteins was normalized to cell number in culture at the time of media collection and presented as mean ± S.D., the expression of these genes were not detected in RNAseq.


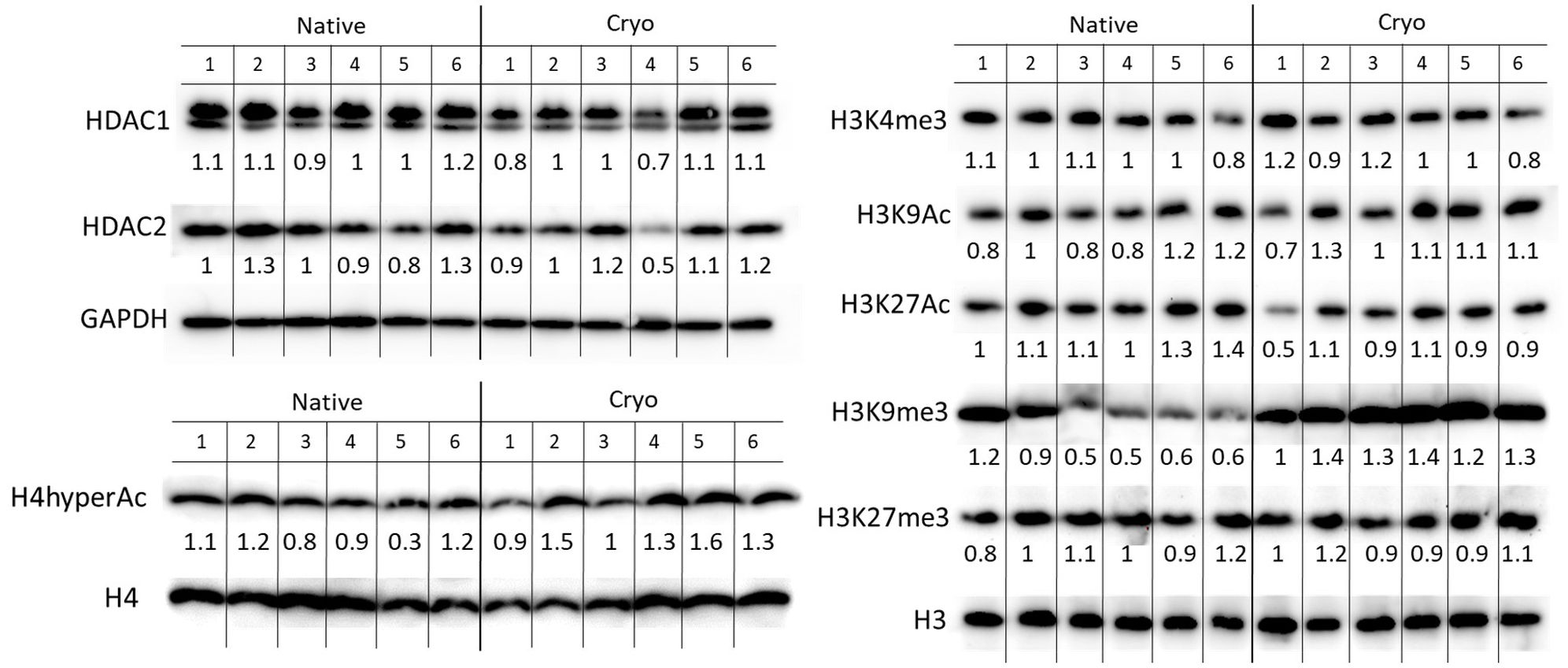


**Supplementary Figure 3.** Representative images of the protein levels in hPMSCs. Protein levels associated with epigenetic regulation were assessed in Native and Cryo cells using Western Blot analysis and relative band intensity was calculated using ImageJ software. Three different proteins GAPDH, H3 and H4 were selected as loading controls: GAPDH for HDAC1 and HDAC2 normalization; H3 – for histone H3 modifications, and H4 for histone H4 modification. Values under the bands represent relative band intensity (*n*=6).


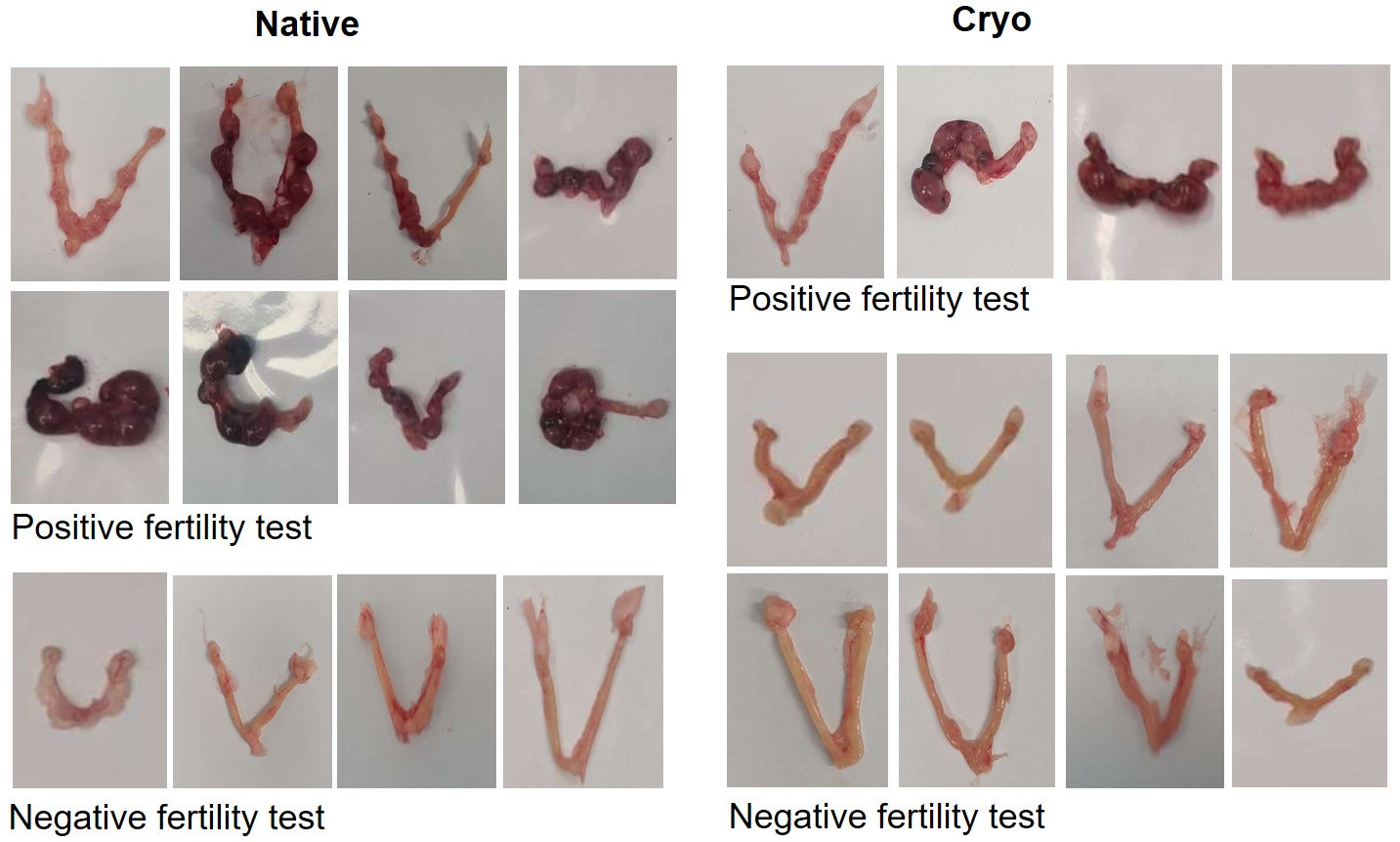


**Supplementary Figure 4.** hPMSCs effect on fertility in the POF mice model. Raw images of uterine horns of POF mice after hPMSCs (Native and Cryo) treatment and after further breeding with a male for two weeks.
