# Supplementary material for: Effects of human placenta cryopreservation on molecular characteristics of placental mesenchymal stromal cells
Source: Front Bioeng Biotechnol. 2023 Apr 13;11:1140781. doi: 10.3389/fbioe.2023.1140781 (PMC10133466; doi:10.3389/fbioe.2023.1140781)
Supplement: Supplementary file 3 [file Table2.DOCX]

Supplementary Material

Effects of Human Placenta Cryopreservation on Molecular Characteristics of Placenta Mesenchymal Stromal Cells

Rūta Navakauskienė^1^*, Deimantė Žukauskaitė^1^, Veronika Viktorija Borutinskaitė^1^, Tetiana Bukreieva^2,3^, Giedrė Skliutė^1,4^, Elvina Valatkaitė^1^, Aistė Zentelytė^1^, Lina Piešinienė^4^, Volodymyr Shablii^2,3^*

*** Correspondence:** Rūta Navakauskienė: [ruta.navakauskiene@bchi.vu.lt](mailto:ruta.navakauskiene@bchi.vu.lt); Volodymyr Shablii: [shablii@stemcellclinic.com.ua](mailto:shablii@stemcellclinic.com.ua)

**Supplementary Table 1.** List of primers sequences.

| **Name** | **Primer sequence** | **Product size, bp** |
| --- | --- | --- |
| *GAPDH* | F: GTGAACCATGAGAAGTATGACAAC | 123 |
|  | R: CATGAGTCCTTCCACGATACC |  |
| *RPL13A* | F: GTTGATGCCTTCACAGCGTA | 128 |
|  | R: AGATGGCGGAGGTGCAG |  |
| *PPARG* | F: CGACCAGCTGAATCCAGAGT | 554 |
|  | R: TTGCCAAGTCGCTGTCATCT |  |
| *OPN* | F: GAAGTTTCGCAGACCTGACAT | 91 |
|  | R: GTATGCACCATTCAACTCCTCG |  |
| *ALP* | F: AGCCCTTCACTGCCATCCTGT | 68 |
|  | R: ATTCTCTCGTTCACCGCCCAC |  |
| *COL2A1* | F: GGCAATAGCAGGTTCACG | 79 |
|  | R: CGATAACAGTCTTGCCCCACTTA |  |

**Supplemental Table 3.** List of primers sequences used for MeDIP.

| **Region** | **Primer sequence** |
| --- | --- |
| *WNT4* promoter | F: TCCTCCCAATCACAGCGTCT |
|  | R: GTGGGAATCCGAAACCTCGC |
| *WNT4* exon | F: AACTGCTCCACACTCGACTC |
|  | R: TGCTCACGAGCGTCTCATTT |
| *HAND2* promoter | F: TGAGGAGGTAGCCAATCCTG |
|  | R: AGGGCCGCTCGGGTTAATA |
| *HAND2* exon | F: CAGCTACATCGCCTACCTCA  R: TCTCCTCTTTCACGTCGGTC |
| *FOXO1* promoter | F: GCTCTGCTGCTCCGTAGTAA |
|  | R: TCTCTCGCCTTCTCAGTGTT |
| *FOXO1* exon | F: TGGAGTACATTTCGCCCTCG |
|  | R:AGTAGAGGCCATCTTTGCGG |
| *HOXA10* promoter | F:ATGTGGTCGTAAACCCGTCC |
|  | R:CAGCCCGCTGCTATTGAGAT |
| *HOXA10* exon | F:AAGAGTGGTCGGAAGAAGCG |
|  | R:GACGCTGCGGCTAATCTCTA |
| *STAT5A* promoter | F: GGGGCGCTGGCTAGTTTAT |
|  | R: AAACTCAACCCTGACGGAGG |
| *STAT5A* exon | F: CACCCGCAAGTAATTGTGCC  R: TGGATGCAAGGACAAAGCGG |
| *GAPDH* promoter | F: TACTAGCGGTTTTACGGGCG  R: GGCTGCGGGCTCAATTTATAG |
